# White matter microstructure of the cingulum bundle and the uncinate fasciculus in primary and secondary callous-unemotional traits

**DOI:** 10.1007/s00787-025-02806-6

**Published:** 2025-07-02

**Authors:** Luisa Schalk, Arjun Sethi, Melanie C. Saam, Itziar Flamarique, Jilly Naaijen, Andrea Dietrich, Pieter J. Hoekstra, Tobias Banaschewski, Pascal-M Aggensteiner, Nathalie Holz, Sarah Baumeister, Roger Borràs, Mireia Rosa-Justicia, Paramala Santosh, Ilyas Sagar-Ouriaghli, Celso Arango, María José Penzol, Daniel Brandeis, Susanne Walitza, Julia E. Werhahn, Barbara Franke, Jeffrey C. Glennon, Marcel P. Zwiers, Ulrike M. E. Schulze, Josefina Castro-Fornieles, Jan K. Buitelaar, Michael C. Craig

**Affiliations:** 1 Department of Forensic and Neurodevelopmental Sciences, Institute of Psychiatry, Psychology and Neuroscience, King’s College, London, UK; 2 Department of Child and Adolescent Psychiatry/Psychotherapy, University Hospital, University of Ulm, Ulm, Germany; 3 Department of Child and Adolescent Psychiatry and Psychology, Clinic Institute of Neurosciences, Hospital Clínic de Barcelona, 2017SGR881, IDIBAPS, CIBERSAM, Barcelona, Spain; 4 Department of Cognitive Neuroscience, Donders Institute for Brain, Cognition and Behavior, Radboud University Medical Center, Nijmegen, The Netherlands; 5 Donders Institute for Brain, Cognition and Behavior, Centre for Cognitive Neuroimaging, Radboud University, Nijmegen, The Netherlands; 6 Department of Child and Adolescent Psychiatry, University of Groningen, University Medical Center, Groningen, The Netherlands; 7 Department of Child and Adolescent Psychiatry and Psychotherapy, Medical Faculty Mannheim, Central Institute of Mental Health, Heidelberg University, Mannheim, Germany; 8 Department of Child and Adolescent Psychiatry and Psychology, Clinic Institute of Neurosciences, Hospital Clinic de Barcelona, IDIBAPS, Barcelona, Spain; 9 Department of Child and Adolescent Psychiatry, Institute of Psychiatry, Psychology and Neuroscience, King’s College, London, UK; 10 Department of Child and Adolescent Psychiatry, Institute of Psychiatry and Mental Health, CIBERSAM, ISCIII, School of Medicine, Hospital General Universitario Gregorio Marañón, Universidad Complutense, IiSGM, Madrid, Spain; 11 Department of Child and Adolescent Psychiatry and Psychotherapy, University Hospital of Psychiatry Zurich, University of Zurich, Zurich, Switzerland; 12 Neuroscience Center Zurich, University and ETH Zurich, Zurich, Switzerland; 13 Department of Human Genetics, Donders Institute for Brain, Cognition and Behavior, Radboud University Medical Center, Nijmegen, The Netherlands; 14 Department of Psychiatry, Donders Institute for Brain, Cognition and Behavior, Radboud University Medical Center, Nijmegen, The Netherlands; 15 Karakter Child and Adolescent Psychiatry University Center, Nijmegen, The Netherlands; 16 Department of Child and Adolescent Psychiatry and Psychology, Department of Medicine, Institute of Neuroscience, 2021SGR01319, Institut Clinic de Neurociències, Hospital Clínic de Barcelona, IDIBAPS, CIBERSAM, University of Barcelona, Barcelona, Spain; 17 German Center for Mental Health (DZPG), Partner Site Mannheim-Heidelberg-Ulm, Germany; 18 Present address: Centre for Neuroimaging Sciences, Institute of Psychiatry, Psychology and Neuroscience, King’s College, London, UK; 19 Present address: UCD Conway Institute of Biomolecular and Biomedical Research, School of Medicine, University College Dublin, Dublin, Ireland

**Keywords:** Disruptive behaviour disorders, Callous-unemotional traits, Internalising, Uncinate fasciculus, Cingulum bundle

## Abstract

Callous-unemotional (CU) traits appear to be linked to differences in neural networks, including the uncinate fasciculus (UF) and cingulum bundle (CB). It has recently been suggested that CU traits are comprised of primary and secondary subtypes based on the relative co-occurrence of internalising symptoms. Here, we explored the white matter microstructure of these subtypes. White matter microstructure of the UF and CB was analysed in youths (aged 8–18; *n* = 121; females = 31) recruited through Europe-based multisite studies. Our sample comprised a control (*n* = 53) and a case group with disruptive behaviour disorders (DBDs) (*n* = 68) with low and high levels of CU traits. The case group was further split into primary (*n* = 49) and secondary (*n* = 14) subgroups. Compared to the control group, the case group showed decreased mean diffusivity in the right ventral CB (*p =*.023, uncorrected). Within our case group, higher scores on the ‘unemotional’ subscale of the Inventory of Callous Unemotional Traits were associated with decreased axial diffusivity (*p* =.02, uncorrected) and mean diffusivity (*p =*.013, uncorrected) in the dorsal CB in youths with primary CU traits, but the opposite in those with secondary CU traits. *Post hoc* analyses found that the left hemisphere drove these effects. The relationship between CU traits and dorsal CB microstructure appears dependent on the presence of internalising symptoms and CU traits subcomponents. A fractionation may lead to a better understanding of the aetiology of DBDs and more focused treatment options.

**Supplementary Information:**

The online version contains supplementary material available at 10.1007/s00787-025-02806-6.

## Introduction

Disruptive behaviour disorders (DBDs) are among the most common child and adolescent disorders and include conduct disorder (CD) and oppositional defiant disorder (ODD). They are characterised by antisocial behaviour and are predictive of substance misuse and other mental and physical health conditions in later life [1, 2]. Further, it has been estimated that about a quarter of children diagnosed with CD will fulfil the criteria for antisocial personality disorder in adulthood [3].

DBDs are highly heterogeneous with many different combinations of antisocial behaviours possible [4]. This is important as some combinations are understood to form specific phenotypic dimensions, associated with differential prognoses and resilience to change, compared to others. One dimension of antisocial behaviour among youths that has consistently been associated with a poorer outcome is the DSM-5 specifier ‘with limited prosocial emotions’, or callous-unemotional (CU) traits, which are based on four items, lack of remorse, lack of empathy, being unconcerned about performance and shallow affect [1, 5]. High levels of CU traits are, for example, associated with impaired recognition of punishment and fear cues [6], resistance to treatment, more consistent problematic behavioural patterns, and extreme forms of aggression [7].

Brain imaging studies of individuals with varying severity of CU traits have identified structural and functional differences in brain areas associated with emotion processing. For instance, in response to fearful faces, functional brain imaging studies have consistently found hypoactivation of the amygdala in DBD children with high versus low CU traits or typically developing children [8, 9]. Also, studies of white matter connectivity have reported that these groups are associated with microstructural changes. Changes in white matter tracts associated with empathy and socioemotional processing have generated significant attention, as deficiencies in these domains have been implicated in CU traits. Two major pathways recognised as important in CU traits are the uncinate fasciculus (UF) and the right cingulum bundle (CB), connecting prefrontal and limbic regions [10]. Microstructural changes in these pathways can be quantified using four diffusion tensor imaging measures: fractional anisotropy, axial diffusivity, radial diffusivity, and mean diffusivity. Fractional anisotropy represents the directional restriction of water diffusion along white matter tracts and is believed to indicate axonal density [11]. Axial diffusivity measures diffusion parallel to the axons and is hypothesized to reflect axonal integrity. Radial diffusivity describes the diffusion perpendicular to the axon and is associated with myelin integrity. Lastly, mean diffusivity represents the overall diffusion of water molecules in a voxel across all directions and is influenced by factors such as cellularity [12].

Microstructural findings include a negative correlation between CU severity and radial diffusivity in the left dorsal cingulum bundle [13] and a negative correlation with mean diffusivity and radial diffusivity in the left UF [14], pointing towards greater white matter microstructure. However, there have also been inconsistencies in these, and other, studies. For example, a negative association between CU traits and fractional anisotropy in the bilateral UF has been reported in participants with conduct problems (CP) [15], indicative of reduced white matter microstructure. In addition, diffusion imaging studies have previously described sex-specific associations between white matter microstructure and CD and CU traits. A recent study reported sex-by-diagnosis interactions in the left UF. Female youths with CD showed higher mean diffusivity compared to typically developing youths, whereas male youths with CD presented with lower mean diffusivity compared to typically developing males. In line with this finding, a study found CU traits in female youths with CD to be negatively associated with axial diffusivity in the left UF and positively in male youths with CD [16]. Another study reported that females with CD showed higher fractional anisotropy compared to female controls in the right retrosplenial cingulum, whereas males with CD presented with lower fractional anisotropy in comparison to controls ​ [17]. These findings emphasise the importance of incorporating sex as a variable in studies of CU traits to explore sex-specific differences in white matter microstructure.

In addition to sex differences, putative explanations for these inconsistencies include heterogeneity of the CU construct involving various subdimensions and/or a lack of power to explore this construct further. CU-traits can be understood as a multi-dimensional construct, consisting of three behavioural components, ‘callousness’, ‘uncaring’, and ‘unemotional‘. ‘Callousness’ refers to callous attitudes regarding others, including a lack of empathy and guilt. The ‘uncaring’ dimension describes an absence of care for performance and others. The ‘unemotional’ component captures a lack of emotional expression [18]. Support for the three-dimensionality of CU-traits includes a study on the association between grey matter volume (GMV) and CU traits in incarcerated adolescents [19]. This found a negative association between callousness and GMV in, for example, the amygdala, ventral anterior cingulate cortex, and medial orbitofrontal cortex (mOFC), and a positive correlation between the uncaring subdimension and GMV in the rostral ACC and right mOFC. No significant correlation was found between the unemotional subdimension and GMV in any of the regions of interest. Investigations of white matter microstructure associated with the CU traits subdimensions remain limited. However, one study that examined microstructure in male and female youths with conduct disorder found the ‘callousness’ subcomponent to be predictive of group differences in radial diffusivity in the bilateral anterior thalamic radiation and axial diffusivity in the corpus callosum [20].

Moreover, it has been suggested that individuals with CU traits can be fractioned into primary and secondary subtypes. The emotional deficits observed in individuals with primary CU traits are understood to have a biological basis (e.g., a greater genetic loading). Conversely, pathogenic environmental factors, such as early traumatic experience, have been proposed to lead to secondary CU [21–23]. Primary and secondary CU traits have been operationalised based on the relative co-occurrence of internalising symptoms (e.g., depressive, somatic, and anxiety symptoms) [21, 24]. Individuals with primary CU traits are characterised by low levels of internalising symptoms, whereas those with secondary CU have high levels of internalising symptoms. In addition to accounting for inconsistencies in the literature, it has been suggested that these subtypes may modulate treatment response and developmental outcomes in adults [23, 25]. For example, a recent longitudinal study found that by age 25 years, individuals with secondary CU traits exhibited greater externalizing behaviours, were more likely to have been involved in violent crimes and had engaged in riskier sexual behaviour than individuals with primary CU [25]. While there is considerable evidence supporting the existence of primary and secondary CU traits, further research is needed to determine whether they are linked to distinct neural correlates.

The present study aims to explore the white matter microstructure implicated in subtypes of antisocial behaviour. Considering the potential significance of fractionation in DBDs, we investigated the relationship between CU trait subdimensions, primary and secondary CU traits, and the white matter microstructure of the CB and UF in male and female children and adolescents. We predicted there would be significant differences in the white matter microstructure of the UF and dorsal CB in cases versus controls. We also hypothesised that in cases, these microstructural differences would (i) correlate with the severity of CU traits and be modulated differently by (ii) primary versus secondary CU traits and (iii) the uncaring, callousness, and unemotional CU subdimensions. Given the inconsistencies in the literature, we did not specify the directionality of the hypothesised differences.

## Methods

### Participants

Data was derived from a multisite project called Multidisciplinary Approaches to Translational Research in Conduct Syndromes (MATRICS) and the EU-Aggressotype project. Participants were recruited from (boarding) schools, resident hospitals, and ambulatories across nine European sites. Our initial sample consisted of 245 participants aged 8 to 18 years. Cases (*n* = 148) were defined by a clinical diagnosis of ODD or CD or a T-score > 70 on the aggression or rule-breaking behaviour subscale (see [7, 26]) of the Child Behaviour Checklist (CBCL; [27]). Participants prescribed medication had been on a stable dose for at least two weeks. Exclusion criteria included a history of a neurological disorder, a learning disability (IQ < 80), or a DSM-5 based diagnosis of major depressive disorder, bipolar disorder, or psychosis, to minimize heterogeneity within the sample due to putative neurobiological and microstructural changes associated with these diagnoses. In the control group (*n* = 97) additional exclusion criteria included scores above clinical threshold on the Kiddie Schedule for Affective Disorders & Schizophrenia (K-SADS) [28] and/or the CBCL [29]. Participants in both groups were also excluded due to missing diffusion tensor imaging (DTI) data (*n* = 25), and/or specific motion criteria [i.e., head motion parameters ≥ 2 SDs away from the mean, ≥ 10 diffusion weighted images (DWIs) less than the total, and/or > 10 contiguous outliers in one slice other than the first eight slices or the final slice] (*n* = 32). Further, 3/9 sites were excluded due to insufficient data quality (see 2.4. Data processing). The exclusion of data based on these criteria yielded a final sample of 121 individuals (68 cases; 31 females; M_age_=13.38; SD_age_=2.6) of White ethnicity. Ethical approval for the project was obtained by each of the nine research institutes independently. Participation was voluntary and written informed consent was obtained before the onset of the study.

### Clinical measures

The semi-structured interview K-SADS was used to confirm diagnoses of ODD and CD and to identify comorbid ADHD, yielding a total score for ADHD symptomatology [28]. K-SADS assessments were conducted independently with the participants and the parents or primary caregivers by trained mental health professionals or trained and supervised research team members. The parent-rated Inventory of Callous Unemotional Traits (ICU, Cronbach’s α =.74 to.85) [30], consisting of three subscales, was applied to assess CU traits and their subcomponents, namely uncaring, callousness, and unemotional [31]. Primary and secondary CU traits were operationalised based on the T-scores of the broadband internalising problem subscale of the caregiver report CBCL (including ‘withdrawn’, ‘somatic complaints’ and ‘anxiety/ depressed problems’ items, Cronbach’s α = 0.72 to 0.88) [32]. A cut-off of ≤ 70 was applied to categorise an individual into a primary CU trait group (low internalising symptoms) and > 70 to categorise an individual into a secondary CU trait group (high internalising symptoms) [24].

### Image acquisition

Imaging measurements were obtained at a separate appointment. Imaging took place at nine European sites using Siemens, Philips, and GE 3-T systems, providing diffusion magnetic resonance imaging (MRI) and T1-weighted MRI data (see supplementary information Table A.1 for scan-site parameters).

### Data processing

#### Image processing and analysis

**Fig. 1:**
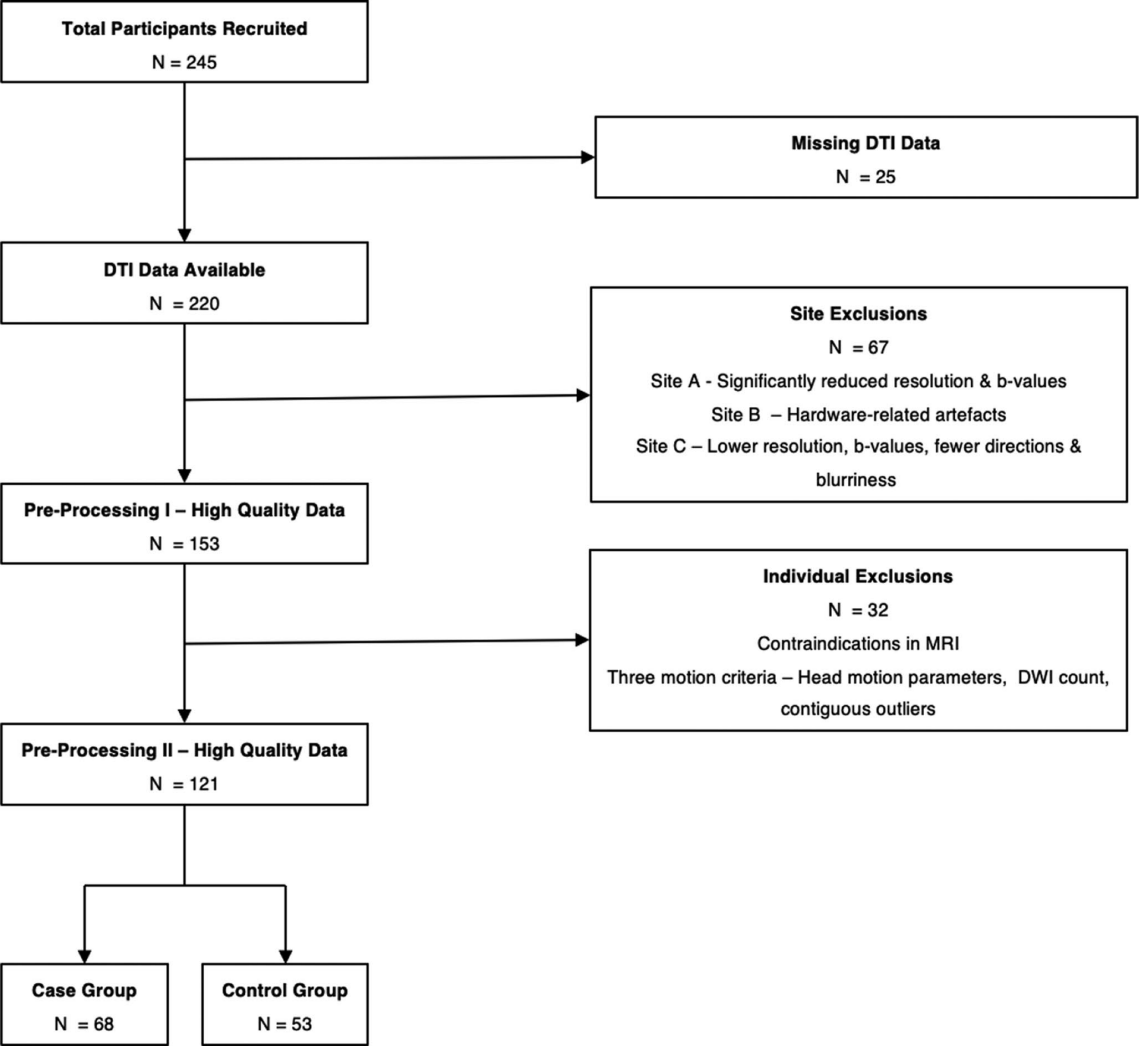
Flow chart of data exclusion and inclusion

DTI data was de-noised and corrected for Gibb’s ringing artefacts using TORTOISE [33]. Data outlier regeneration, slice-to-volume correction and motion correction were performed using FSL’s Diffusion Toolbox (FMRIB’s Software Library). Following pre-processing, three sites with insufficient data quality were excluded. One site was excluded due to significantly reduced resolution and b-values; another due to hardware-related artefacts; and a third site because of lower resolution, b-values, fewer directions, and blurriness (see Fig. 1). The software Startrack (https://www.mr-startrack.com/) was used to fit the diffusion tensor and to perform whole-brain tractography (FA threshold: 0.20, step size:1 mm, Angle threshold:35) for each subject. Individual datasets were then combined using the software MegaTrack to create a single tractography representing the whole dataset [34].

#### Tractography dissection

Virtual dissections of the UF and CB were performed at the whole dataset level, for both hemispheres, using TrackVis software [35].

**Fig. 2:**
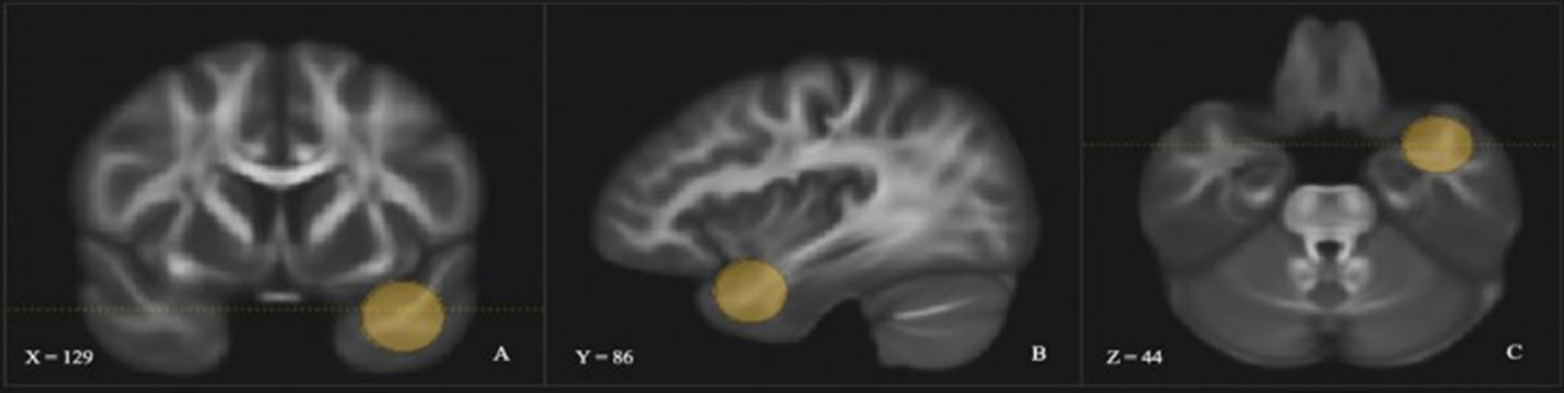
Coronal (**A**), sagittal (**B**), and axial (**C**) view of the left UF anterior temporal ROI within the data set. Depicted in one hemisphere only for illustrative purposes. Does not depict ROIs used to exclude fibres that were independent of the UF

**Fig. 3:**
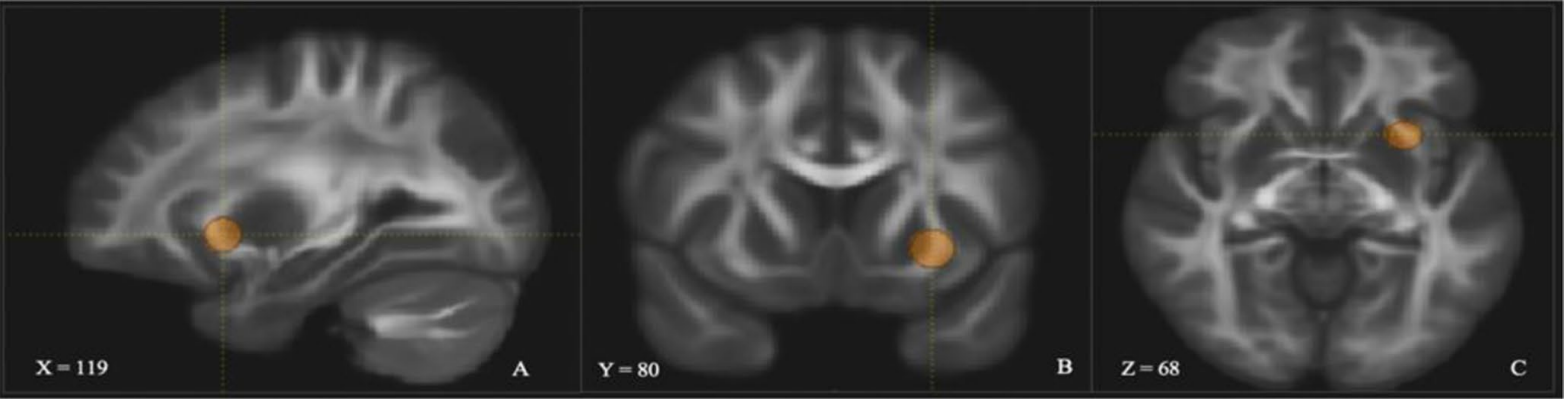
Coronal (**A**), sagittal (**B**), and axial (**C**) view of the left UF medial and lateral orbitofrontal ROI within the data set. Depicted in one hemisphere only for illustrative purposes. Does not depict ROIs used to exclude fibres that were independent of the UF

**Fig. 4:**
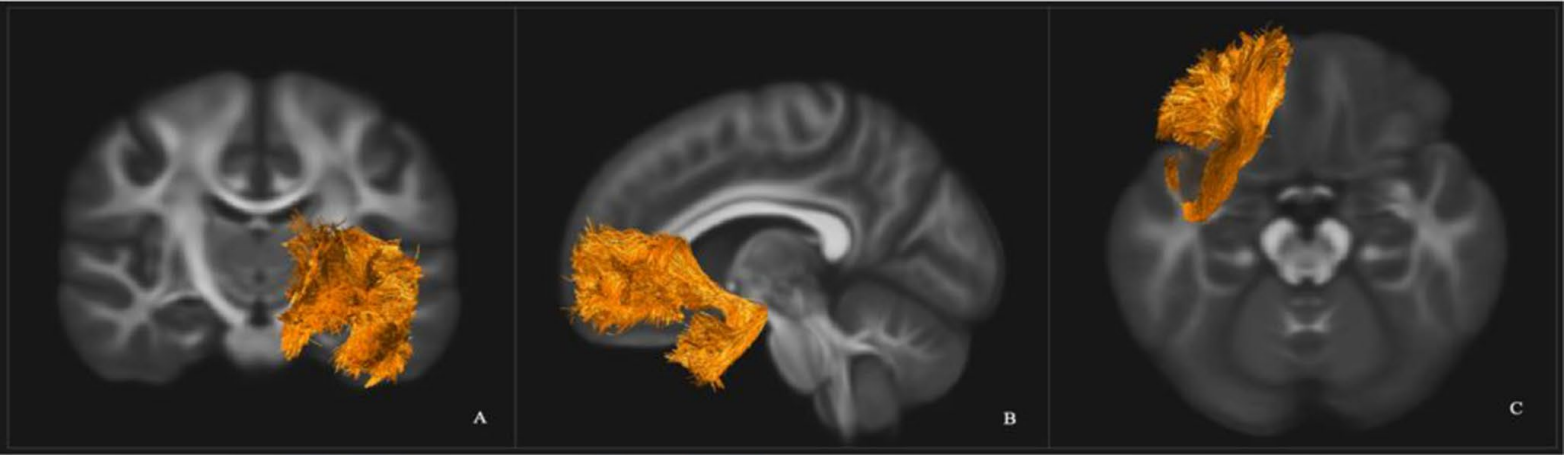
Coronal (**A**), sagittal (**B**), and axial (**C**) view of the left UF. Depicted in one hemisphere only for illustrative purposes

The UF was dissected using two regions of interest (ROI) [36]. The first was in the anterior temporal lobe (see Fig. 2) and the second was placed around the medial and lateral orbitofrontal cortex (see Fig. 3). In addition, several ROIs were used to exclude fibres that were independent of the UF (see Fig. 4).

**Fig. 5:**
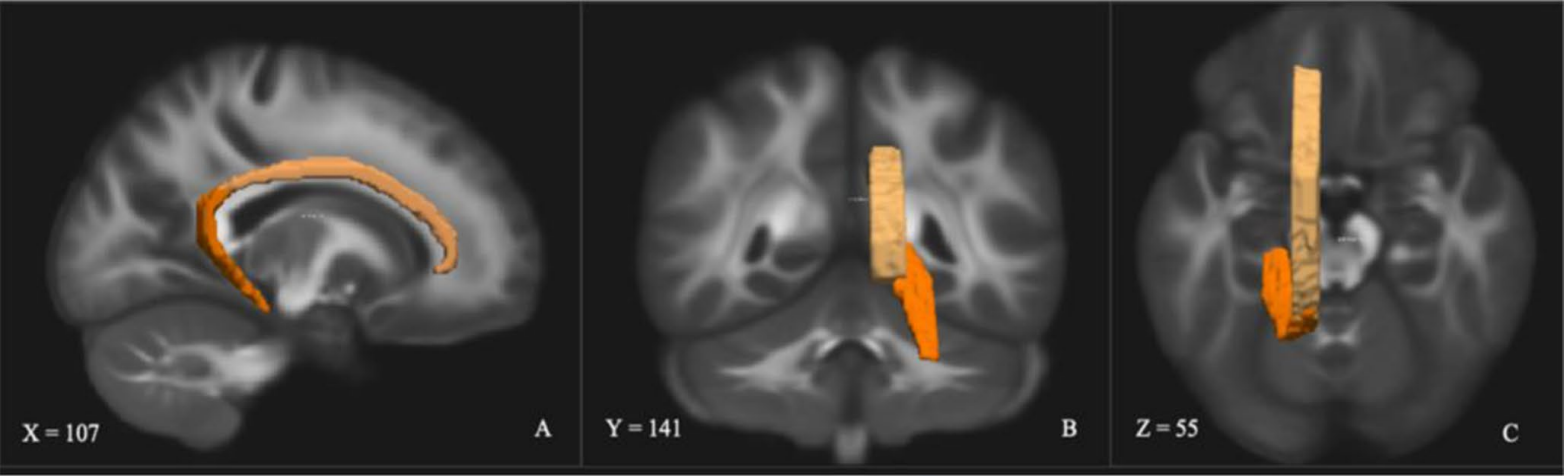
Coronal (**A**), sagittal (**B**), and axial (**C**) view of the dorsal (light orange) and ventral (dark orange) CB ROIs within the data set. Depicted in one hemisphere only for illustrative purposes. Does not depict ROIs used to exclude fibres that were independent of the CB

**Fig. 6:**
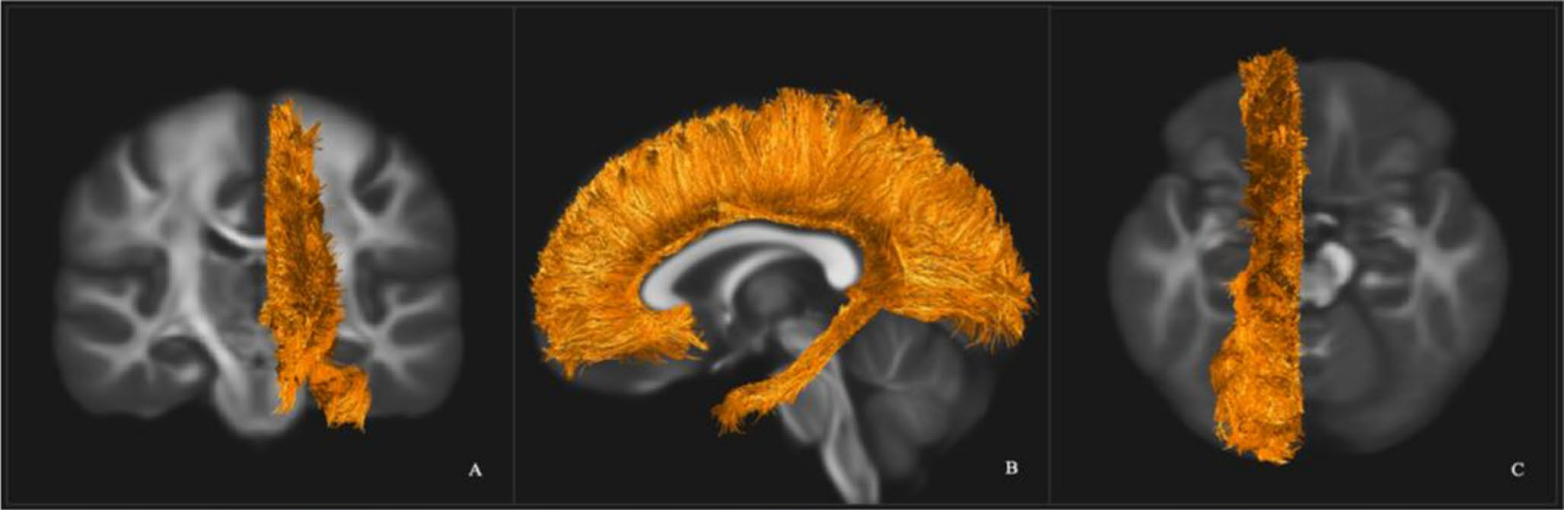
Coronal (**A**), sagittal (**B**), and axial (**C**) view of the left CB. Depicted in one hemisphere for illustrative purposes

The CB was initially identified on the axial plane, following which the white matter pathway was dissected into dorsal and ventral portions based on a two ROI approach. These were defined by anatomical ROIs created from the Human Connectome Project data co-registered to the study-specific Mega Track dataset [37]. The point of the division was defined on the midline slice above the splenium of the corpus callosum (see [38], see Fig. 5). Several ROIs were again used to eliminate fibres that were independent of the CB (see Fig. 6).

Once virtual dissections were completed, MegaTrack was used to extract tract-specific measurements from each subject’s native space data. For each tract, the streamline count, mean values for fractional anisotropy, axial diffusivity, radial diffusivity and mean diffusivity and the tract volume were extracted.

### Statistical analyses

All analyses were performed using R Statistical Software [39]. Descriptive statistics comparing cases and controls were performed for sex, age, IQ, medication, and ADHD symptom count. Clinical information was compared between cases and controls for the continuous measures CU traits and its subscales and the dichotomous diagnosis of ODD and CD and primary and secondary CU traits (as indicated by low and high internalising scores on the CBCL). Linear mixed models were used to analyse the effect of diagnosis and CU traits on the dependent variables fractional anisotropy, axial diffusivity, radial diffusivity, and mean diffusivity, in the UF and the ventral and dorsal CB. The nlme package in R was used to model the data (package ‘nlme’; [40]). Age, sex, IQ, and ADHD symptom count were included as covariates of non-interest. A random intercept model, nesting the subjects in the scan site, was constructed to account for the effects associated with different sites. Group differences were explored by examining the fixed effect of the group, and its interaction with the hemisphere. Given the significant difference in CU traits between cases and controls, analyses on the predictive effect of CU traits were conducted separately in the case group. To investigate the effects of CU traits and their components, scores on ICU and its three subscales were modelled as interaction with hemisphere. Analyses were repeated with internalising symptoms (‘low’ or ‘high’) as an additional variable (see supplementary information Eq. [A.1] for model statements). *Post hoc* analyses were performed splitting by hemisphere. Sensitivity analyses were performed to identify and remove potential outliers. To correct for multiple comparisons (two unique diffusion measures, three white matter tracts, and three tests), an adjusted *alpha* of 0.003 (0.05/15 tests) was applied.

## Results

### Demographics

High-quality data were available for 121 participants (cases = 68, controls = 53) across six sites. Sixty cases were diagnosed with ODD and/or CD with the remaining eight included based on their scores on the aggression or rule-breaking subscale of the CBCL. Internalising data was available for all participants of the control group (*n* = 53) and 63 participants within the case group (*n* = 68; see Table 1). Compared to the control group, the case group was comprised of more males (*p* <.001), lower IQ (*p* <.001) and greater ADHD symptom count (*p* <.001). The groups did not differ significantly in age (0.102).

**Table 1:** Demographic and clinical information of the total sample (*n* = 121) included in the DTI analysis following exclusion

	Cases (*n* = 68)	Controls (*n* = 53)	
	N	N	*p* values
Sex			< 0.001
Female	9 (13.24%)	22 (41.51%)	
Male	59 (86.76%)	31 (58.49%)	
DBDs			
CD	21 (35%)	–	
ODD	39 (65%)	–	
CU Subtypes (CBCL, T score internalising)			
Primary	49 (77.78%)	–	
Secondary	14 (22.22%)	–	
	Mean (SD)	Mean (SD)	
Age	13.03 (2.7)	13.81 (2.4)	0.102
IQ	99.59 (11.9)	107.3 (11.6)	< 0.001
Medication	43 (63.2)	1 (1.9)	< 0.001
ADHD (K-SADS, symptom count)	31.9 (13.2)	5.12 (6.1)	< 0.001
CU (ICU, total)	31.85 (9.1)	22.08 (8)	< 0.001
Uncaring	14.77 (4.9)	11.1 (4.7)	0.001
Unemotional	7.2 (2.9)	5.69 (2.9)	0.053
Callousness	8.8 (4.7)	3.94 (2.9)	< 0.001

CBCL, Child Behaviour Checklist; K-SADS, Kiddie Schedule for Affective Disorders & Schizophrenia; ICU, Inventory of Callous-Unemotional Traits

### Group differences in fractional anisotropy, axial diffusivity, radial diffusivity, and mean diffusivity

**Fig. 7:**
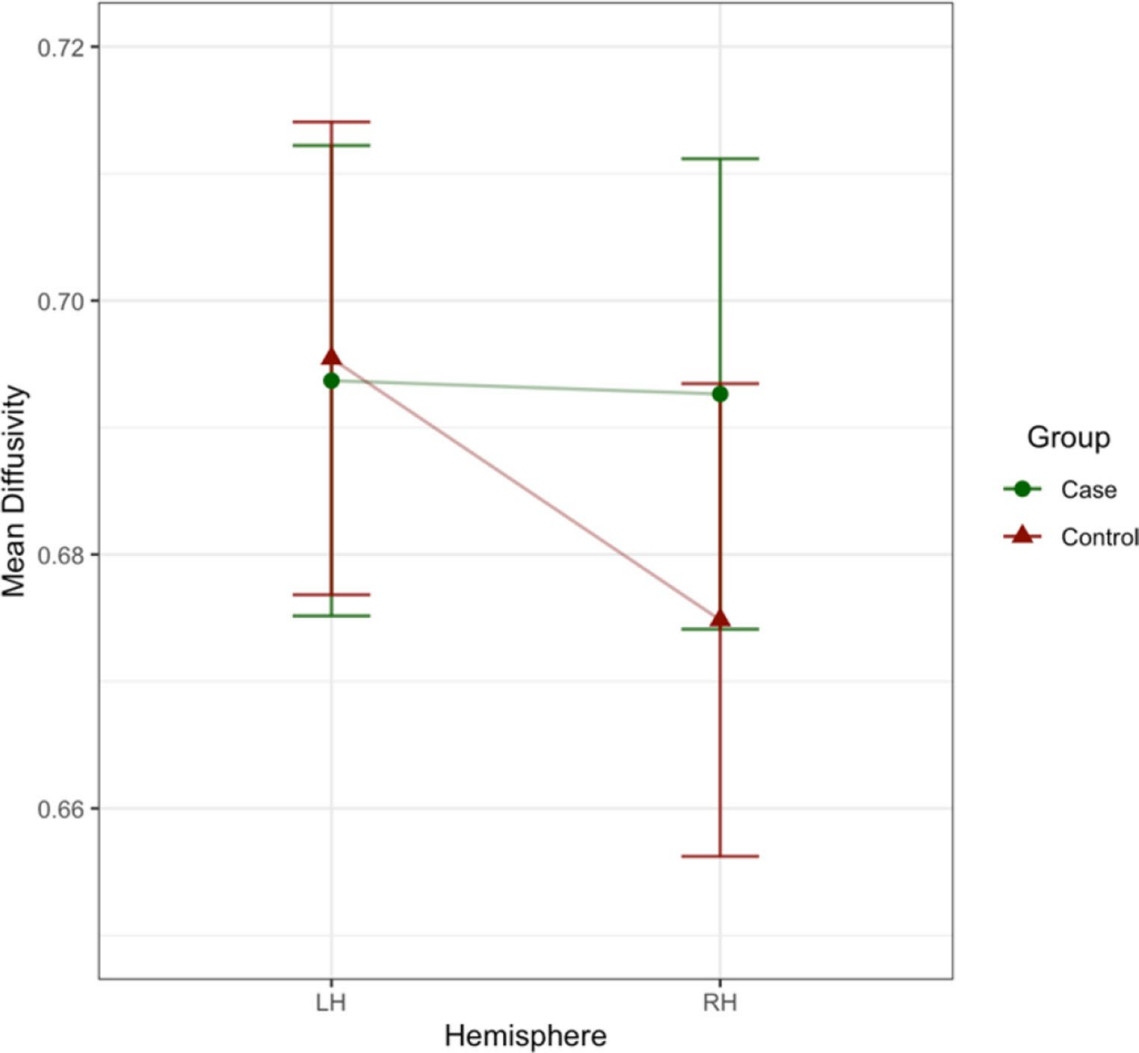
Group differences (case versus control) on mean diffusivity in the ventral CB. The control group showed lower mean diffusivity in the right hemisphere (RH) compared to the left hemisphere (LH) and the case group

Examination of group differences (cases vs. controls) on fractional anisotropy, axial diffusivity, radial diffusivity, and mean diffusivity showed a significant two-way interaction effect of ‘group and ‘hemisphere’ on ventral cingulum mean diffusivity [b=-0.018, t (99.33) = -2.31, *p* =.023, see supplementary information Table B.1 for effects]. Investigation of the interaction plot suggested that the control group had significantly decreased mean diffusivity in the right ventral CB compared to the case group (see Fig. 7). After correction for multiple comparisons, the effect remained as a trend. There were no effects of ‘group’ observed for any of the DTI indices (fractional anisotropy, axial diffusivity, radial diffusivity, mean diffusivity) in the UF or dorsal CB.

### Association between CU traits subscales and fractional anisotropy, axial diffusivity, radial diffusivity, and mean diffusivity within the case group

Examination of the effect of CU traits and its three subscales, and fractional anisotropy, axial diffusivity, radial diffusivity, and mean diffusivity on the dorsal CB, the ventral CB and the UF did not yield any significant effects (see supplementary information Table B.2 to B.13 for effects).

### Association between CU traits subscales and fractional anisotropy, axial diffusivity, radial diffusivity, and mean diffusivity within the case group accounting for differences among primary and secondary CU traits

**Fig. 8:**
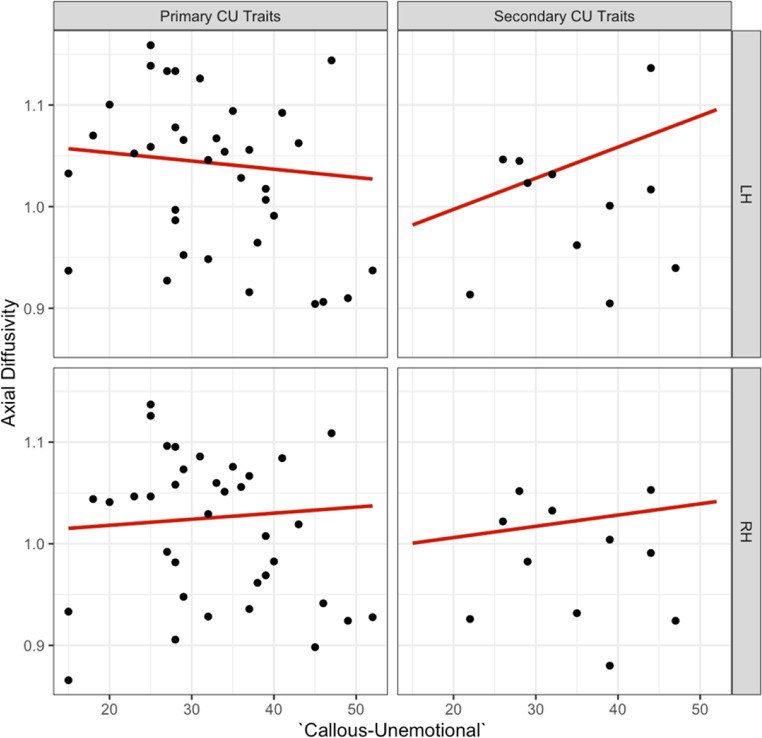
Association between ‘callous-unemotional’, primary versus secondary CU traits and left versus right hemispheres for axial diffusivity in the dorsal CB

Since the neural correlates of primary and secondary CU traits have been found to differ, we included ‘internalising’ symptoms (‘low’ or ‘high’) in the analyses, to assess whether the overall effects of CU traits were masked by primary and secondary subtypes. We observed a significant three-way interaction for ‘CU traits’, ‘internalising’, and ‘hemisphere’ on axial diffusivity in the dorsal CB, which remained as a trend after correcting for multiple testing [b=- 0.003, t (34) = − 2.497, *p* =.018, see supplementary information Table B.14 for effects]. The observed effect of ‘CU traits’ on axial diffusivity was dependent on the level of ‘internalising’ (‘low’ or primary CU traits versus ‘high’ or secondary CU traits, see Fig. 8. *Post hoc* tests indicated a trend for the left hemisphere in driving the effect of ‘callous-unemotionality’ on axial diffusivity [b = 0.004, t (30.6) = 2.77, *p* =.009].

**Fig. 9:**
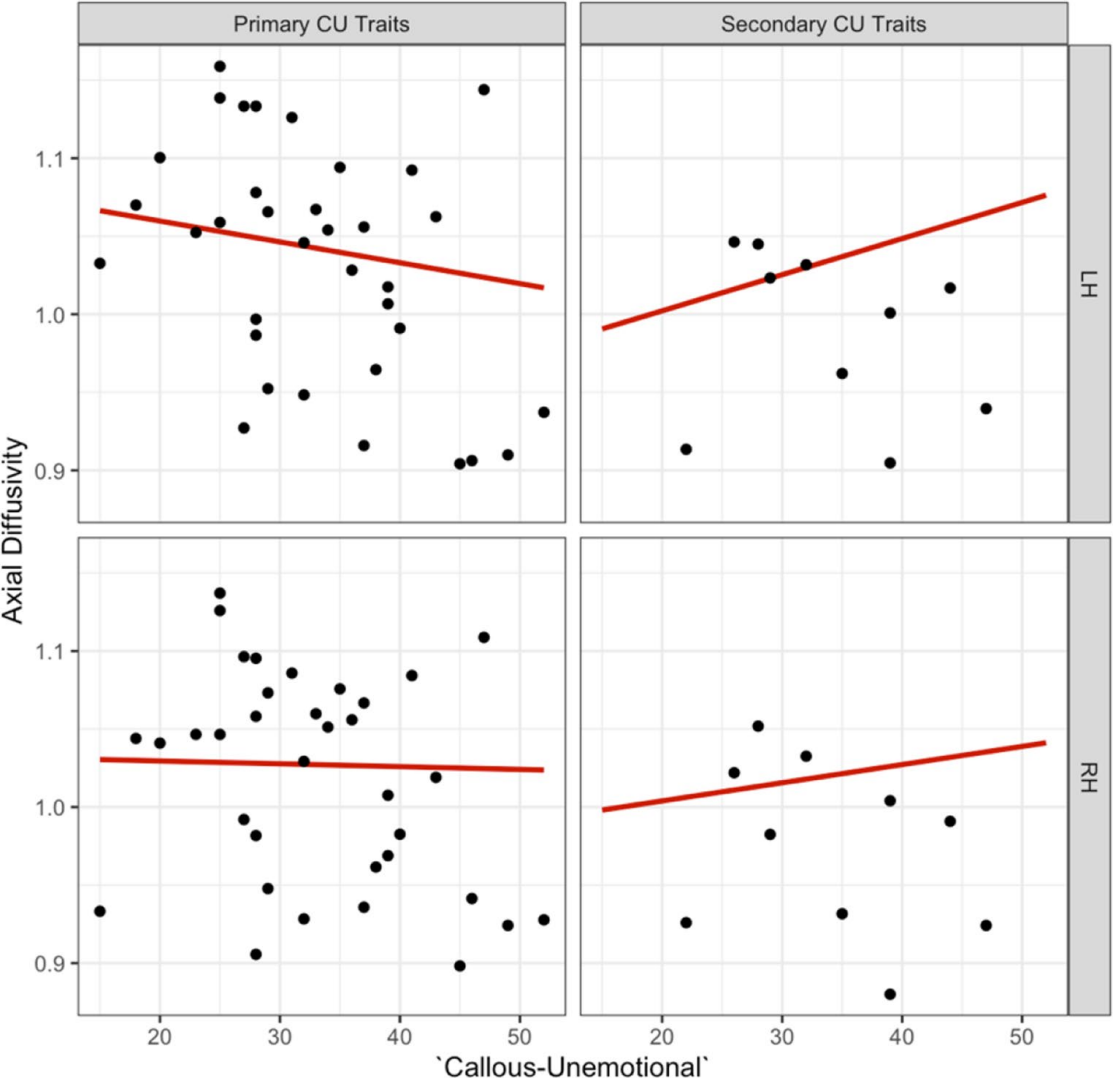
Association between ‘callous-unemotional’, primary versus secondary CU traits and left versus right hemispheres for axial diffusivity in the dorsal CB following outlier removal. The primary subtype showed a decrease in axial diffusivity and the secondary subtype an increase in axial diffusivity in the dorsal CB

A *post hoc* sensitivity analysis (z > ± 2) identified and removed one moderate outlier from each subgroup. Following outlier removal, the previously significant three-way interaction was no longer observed [b = -0.002, t(32) = -1.553, *p* =.13]. However, we observed a trend-level two-way interaction between ‘CU traits’ and ‘internalising’ on axial diffusivity in the dorsal CB [b = 0.004, t(28.36) = 2.466, p = 0.06; see Fig. 9].

**Fig. 10:**
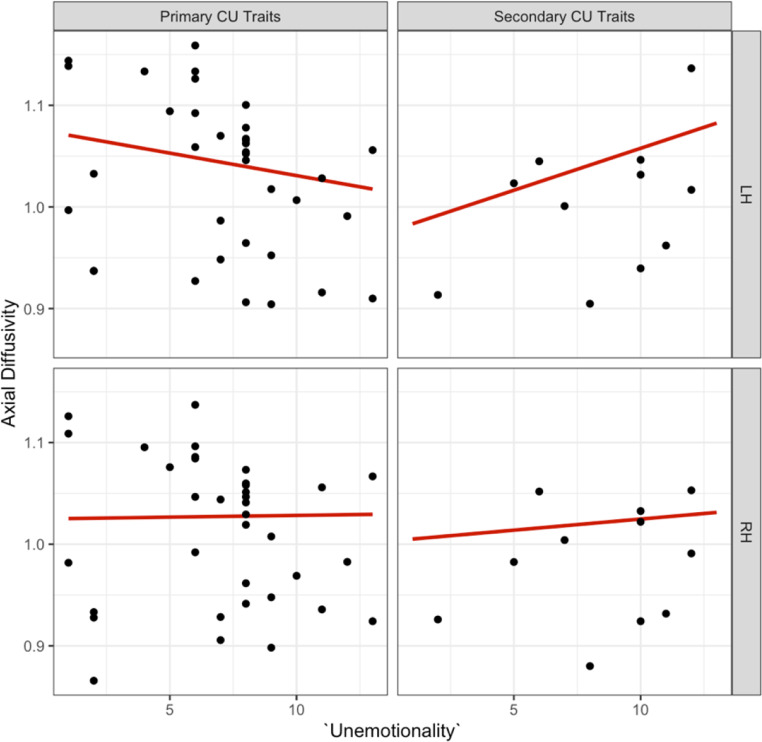
Association between the ‘unemotional’ ICU subscale, primary versus secondary CU traits and left versus right hemispheres for axial diffusivity in the dorsal CB

We observed a significant three-way interaction effect for the ‘unemotional subscale’, ‘internalising’, and ‘hemisphere’ on axial diffusivity in the dorsal CB, which remained significant after correcting for multiple comparisons [b=-0.01, t (34) = -3.28, *p* =.002, see supplementary information Table B.15 for effects]. The effect of ‘unemotionality’ on ‘hemisphere’ depended on the subcategory of CU traits (primary versus secondary). *Post hoc* tests indicated that this interaction was driven by the left hemisphere, such that there was a decrease in axial diffusivity for primary and an increase for secondary CU traits [b = 0.024, t (31.16) = 3.58, *p* =.001, see. Figure 10].

**Fig. 11:**
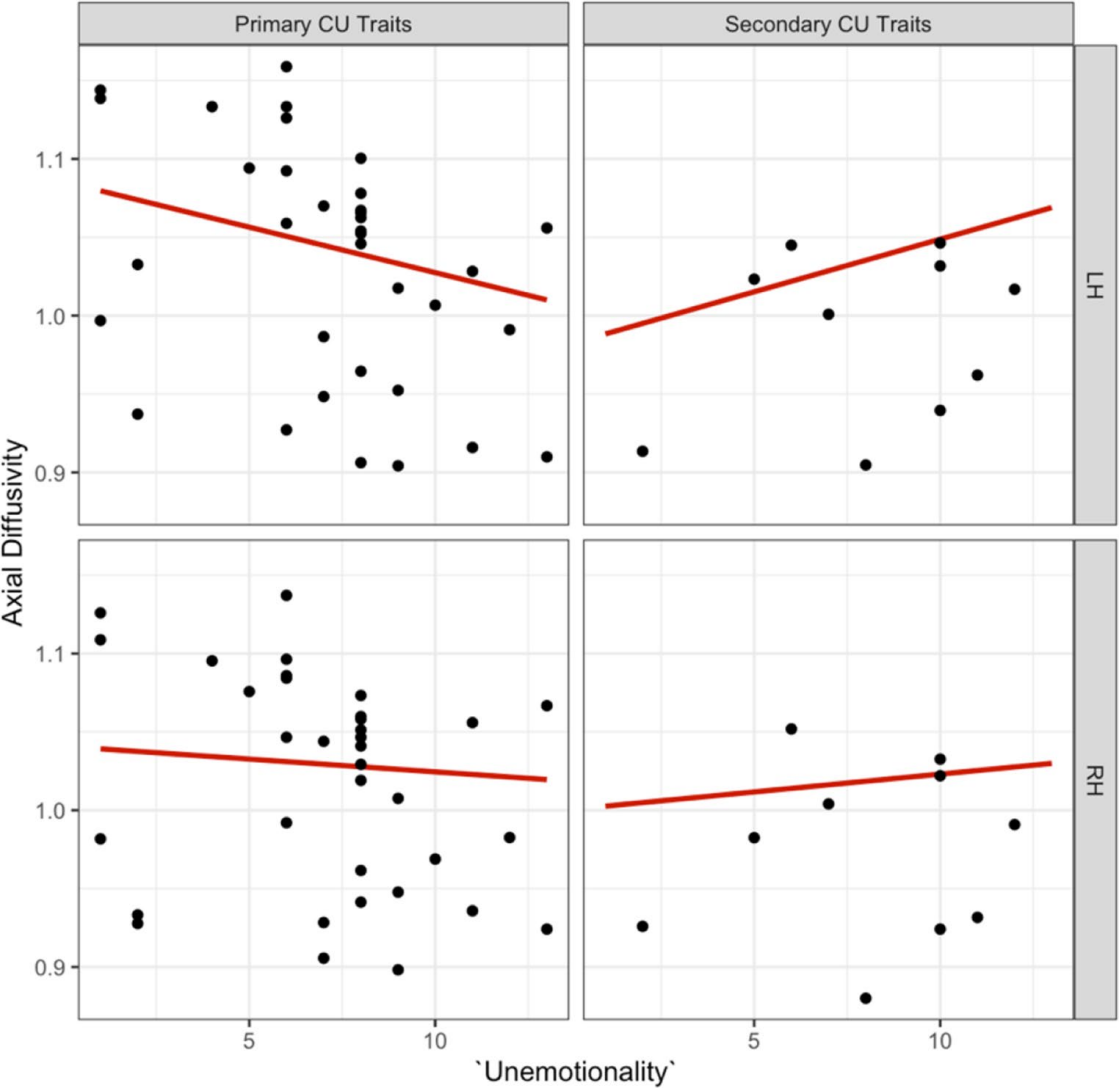
Association between the ‘unemotional’ ICU subscale, primary versus secondary CU traits and left versus right hemispheres for axial diffusivity in the dorsal CB following outlier removal. The primary subtype showed a decrease in axial diffusivity and the secondary subtype an increase in axial diffusivity in the left dorsal CB

After *post hoc* outlier removal, the significant three-way interaction between ‘CU traits’, ‘internalising’, and ‘hemisphere’ on axial diffusivity in the dorsal CB persisted, though it only remained a trend after correcting for multiple comparisons [b=-0.009, t (32) = -2.389, *p* =.02, see Fig. 11]. We did not observe significant three-way interaction effects for the ‘uncaring subscale’, ‘internalising’, and ‘hemisphere’ or the ‘callous subscale’, ‘internalising’, and ‘hemisphere’ on axial diffusivity in the dorsal CB (see supplementary information Figure A.1 and Figure A.2).

**Fig. 12:**
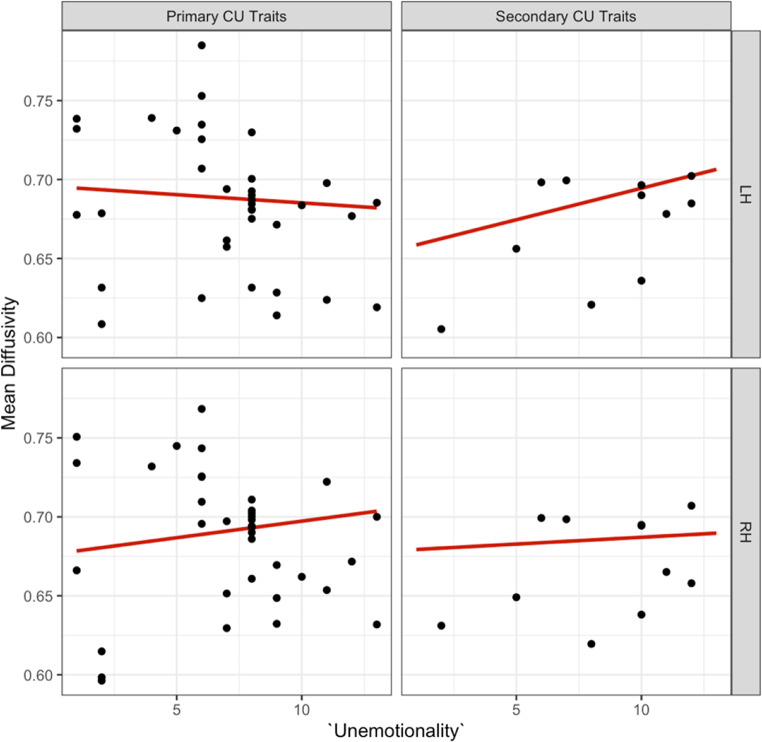
Association between the ‘unemotional’ ICU, primary versus secondary CU traits and left versus right hemispheres for mean diffusivity in the dorsal CB

We also observed a second significant three-way interaction effect for ‘unemotionality’, ‘internalising’ and ‘hemisphere’ on mean diffusivity in the dorsal CB, surviving as a trend after correction for multiple testing [b=-0.006, t (34) = -2.53, *p =*.016, see supplementary information Table C.1 for effects]. Further, the effect of ‘unemotionality’ on ‘hemisphere’ also depended on primary and secondary CU traits. We also found that increased ‘unemotionality’ was associated with decreased mean diffusivity in primary, and increased mean diffusivity in secondary CU traits in the left hemisphere [b = 0.006, t (31.92) = 2.06, *p* =.047, see Fig. 12].

**Fig. 13:**
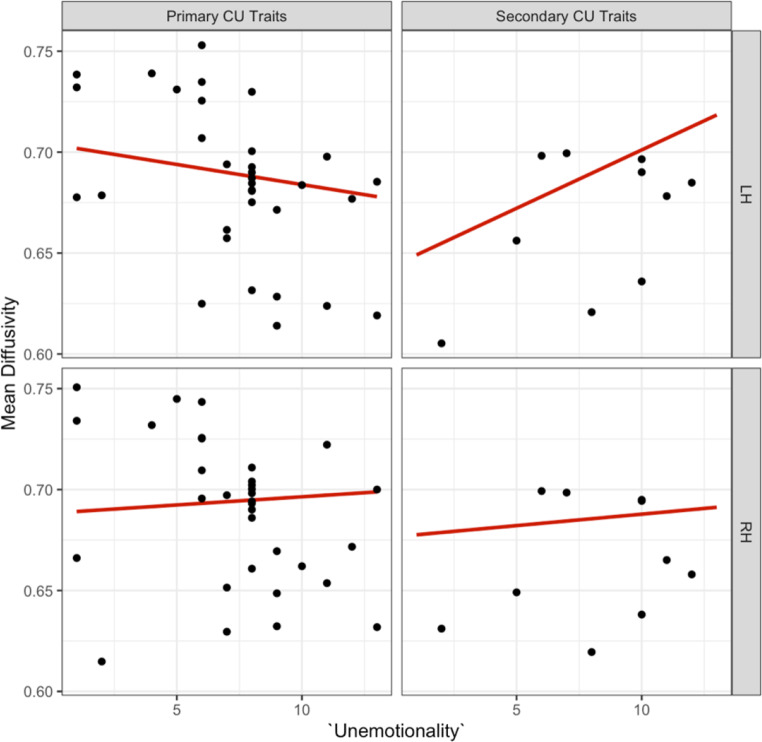
Association between the ‘unemotional’ ICU, primary versus secondary CU traits and left versus right hemispheres for mean diffusivity in the dorsal CB following outlier removal. The primary subtype showed a decrease in mean diffusivity and the secondary subtype an increase in mean diffusivity in the left dorsal CB

A *post hoc* sensitivity analysis (z > ± 2) identified and removed three moderate outliers from the primary subgroup. The previously observed significant three-way interaction on mean diffusivity persisted and remained a trend after correcting for multiple comparisons [b=-0.007, t (30) =-2.640, *p* =.013, see Fig. 13]. We did not observe significant three-way interaction effects for the ‘uncaring subscale’, ‘internalising’, and ‘hemisphere’ or the ‘callous subscale’, ‘internalising’, and ‘hemisphere’ on mean diffusivity in the dorsal CB (see supplementary information Figure B.1 and Figure B.2).

## Discussion

Our multisite study offers preliminary evidence that white matter alterations among youths with DBDs may vary depending on whether CU traits are associated with low or high internalising symptoms, and which subcomponent of the CU construct is being analysed. For example, in youths with primary CU traits, we observed a trend for more severe CU traits to be associated with decreases in axial diffusivity in the dorsal CB, whereas an increase axial diffusivity in the dorsal CB was observed in those with secondary CU traits. These findings hint towards potentially opposing diffusion patterns in the dorsal CB, and consequently, a potential for distinct tract-specific microstructure between primary and secondary CU traits. Our findings are important as prior inconsistencies in this area of research [13, 15] may be understood by a failure to fractionate CU traits into specific subtypes. Further validation of this approach can be found in a study of amygdala activation to threat exposure, which also reported that response was dependent on whether CU traits were primary or secondary [41].

In addition, when CU traits were fractionated into their three subcomponents [6] we found microstructural changes (axial diffusivity /mean diffusivity) in the left dorsal CB were similarly moderated by primary and secondary CU traits for the ‘unemotional’ subcomponent, but not the ‘uncaring’ or ‘callousness’ subcomponents. Notably, concerns have been raised about the construct validity of ICU, specifically regarding weaker associations observed between the ‘unemotional’ subcomponent and the broader CU traits construct when compared to the ‘uncaring’ and ‘callousness’ subcomponents [42]. Given these considerations, it is important to interpret the present finding on unemotionality with caution rather than as definite evidence. The finding, however, is consistent with our previous work indicating distinct seed-based patterns of resting state connectivity for the ‘uncaring’, ’callousness’ and ‘unemotional’ subscales in the same sample [26]. Together, our studies provide preliminary support for the three-dimensionality of the CU construct [18] and highlight a potential importance of fractionating the CU construct into specific subcomponents when studying youths with DBDs.

Previous work reported associations between CU severity and white matter microstructure measures in the left dorsal cingulum bundle [13] and right retrosplenial cingulum [17]. Our findings of differential microstructure of the CB in primary and secondary CU traits offers further support for the dorsal CB as a key structure in DBDs, whereas we do not provide evidence for a role of the ventral CB in CU traits. This observation is in line with previous work, which has emphasised the particular importance of microstructural changes in the dorsal segment of the CB [13]. More generally, the CB represents a white matter tract within the default mode network (DMN), a network integral to emotion processing, executive function and decision-making [38, 43]. In addition to studies describing structural changes within the DMN, studies investigating the functional microstructure have reported, for example, negative associations between CU traits and within-DMN functional connectivity [44]. The observed lateralisation of effects to the left hemisphere in association with the ‘unemotionality’ subdimension aligns with prior research indicating unilateral alterations in white matter microstructure in association with CU traits [13, 14]. Collectively, these findings suggest potential differences in hemispheric brain organisation among youths with DBDs. However, given the preliminary nature of our findings, the interpretability is limited, and further research is required for validation. In particular, the shift from significant findings to trend-level results after sensitivity analyses underscores the vulnerability of our results to moderate outliers, emphasising the importance of methodological consistency and careful consideration of participant variability in multisite neuroimaging studies. Nonetheless, the integration of structural and functional connectivity remains imperative for developing a better understanding of the mechanisms underpinning DBDs, related behavioural expressions and phenotypical fractionation. Validated microstructural changes may function as diagnostic markers for CU traits, thereby enhance prevention and early detection in children and adolescents. Moreover, the identification of distinct neural correlates could inform the development of intervention strategies and treatments targeting alterations to neural networks and specific brain regions.

It was beyond the scope of the current study to explore the aetiology of the differences associated with primary and secondary CU traits. However, primary CU traits have, for example, been associated with specific genetic risk factors [23], whereas secondary CU traits have been more associated with exposure to environmental risk factors, including childhood maltreatment and other traumatic experiences [21, 23]. Limitations of the current study include our sample size. Although this was double compared to prior studies, our final sample size was smaller than that of our investigations on, for example, resting state functional connectivity [26] and brain activity during an emotional face-matching task [8] within the data set. A potential explanation for this is limitations associated with DTI that led to greater exclusion of data. The limitations include susceptibility-induced distortion of images [44] and high sensitivity to motion [46]. A larger dataset might have permitted analysis of any moderating effect of sex on our findings, which was prohibited by the sex bias in our sample (i.e., males = 59, females = 9). Moreover, with a larger study sample, our comparison of the primary (*n* = 48) and secondary (*n* = 14) subtype would be more statistically powerful. Nevertheless, our data on demographics and behavioural measures indicate that our subsample is representative of the overall study sample. A further limitation of our study is the categorical approach to internalising symptoms, which may reduce statistical power compared to dimensional analyses. Future research should consider adopting dimensional approaches, especially in larger and more balanced samples, to further validate and extend our findings.

Despite these shortcomings, our study has highlighted that the current use of the construct of CU traits is likely to be oversimplified. More importantly, the fractionation of DBD youths with high CU traits into primary and secondary subtypes could lead to a better understanding of the neurobiology of this dimension and ultimately to the possibility of more personalised treatments.

## Electronic supplementary material

Below is the link to the electronic supplementary material.

Supplementary Material 1

## Data Availability

No datasets were generated or analysed during the current study.
