# Supplementary material for: White matter microstructure of the cingulum bundle and the uncinate fasciculus in primary and secondary callous-unemotional traits
Source: Eur Child Adolesc Psychiatry. 2025 Jul 2;34(12):4035–51. doi: 10.1007/s00787-025-02806-6 (PMC12743073; doi:10.1007/s00787-025-02806-6)

**SUPPLEMENTARY INFORMATION**

**Table A.1 – Scan-site specific parameters adapted from Craig et al. (2019).**

| Site | Scanner Type | B0 Value | B-Value (s/mm^2^) | DWI Value (Number of Volumes) | Voxel Size (mm) |
| --- | --- | --- | --- | --- | --- |
| Nijmegen | Siemens | 2 | 1500 | 66 | 2 x 2 x 2 |
| Groningen | Siemens | 1 | 1500 | 61 | 2.5 x 2.5 x 2.5 |
| Mannheim | Siemens | 6 | 1500 | 66 | 2 x 2 x 2 |
| Ulm | Philips | 6 | 1500 | 70 | 2 x 2 x 2 |
| London | GE | 6 | 1500 | 66 | 2 x 2 x 2 |
| Barcelona | Siemens | 1 | 1500 | 65 | 2 x 2 x 2 |
| Barcelona | Siemens | 1 | 1500 | 31 | 2 x 2 x 2 |
| Madrid | Siemens | 10 | 1500 | 74 | 2 x 2 x 2 |
| Zurich | Philips | 1 | 1500 | 61 | 2 x 2 x 2 |
| Rome | Siemens | 1 | 1000 | 31 | 2.28 x 2.28 x 2 |

**Eq. (A.1) – Model statements of the statistical analyses.**

The following models were applied to all four white matter microstructure measures (fractional anisotropy, axial diffusivity, radial diffusivity, and mean diffusivity) within the dorsal and ventral cingulum bundle (CB) and the uncinate fasciculus (UF) separately.

*Group effect*

Fractional Anisotropy Dorsal CB ~ ADHD + Age + Sex + IQ + Group * Hemisphere + (1 | Site / ID)

Fractional Anisotropy Dorsal CB ~ ADHD * Hemisphere + Age * Hemisphere + Sex * Hemisphere + IQ * Hemisphere + Group * Hemisphere + (1 | Site / ID)

*Continuous measures of interest (CU traits and subscales) within the case group*

Fractional Anisotropy Dorsal CB ~ ADHD * Hemisphere + Age * Hemisphere + Sex * Hemisphere + IQ * Hemisphere + Continuous Measure of Interest * Hemisphere + (1 | Site/ ID)

Fractional Anisotropy Dorsal CB ~ ADHD + Age + Sex + IQ + Continuous Measure of Interest * Hemisphere + (1 | Site/ ID)

*Continuous measures of interest (CU traits and subscales) accounting for primary and secondary CU traits*

Fractional Anisotropy Dorsal CB ~ ADHD * Hemisphere + Age * Hemisphere + Sex * Hemisphere + IQ * Hemisphere + Continuous Measure of Interest * Hemisphere * Internalising + (1 | Site/ ID)

Fractional Anisotropy Dorsal CB ~ ADHD + Age + Sex + IQ + Continuous Measure of Interest * Hemisphere * Internalising + (1 | Site/ ID)

**Table B.1 – Group differences on Mean Diffusivity in the ventral CB.**

| Type III Analysis of Variance Table with Satterthwaite's method | | | | | | |  |
| --- | --- | --- | --- | --- | --- | --- | --- |
|  | Sum Sq | Mean Sq | NumDF | DenDF F | F value | Pr(>F) |  |
| ADHD | 0.0001344 | 0.0001344 | 1 | 95.833 | 0.4862 | 0.487302 |  |
| Hemi | 0.0000883 | 0.0000883 | 1 | 99.292 | 0.3195 | 0.573212 |  |
| Age | 0.0133966 | 0.0133966 | 1 | 95.793 | 48.4755 | 4.176e-10 | *** |
| Sex | 0.0001316 | 0.0001316 | 1 | 95.963 | 0.4762 | 0.491806 |  |
| IQ | 0.0005309 | 0.0005309 | 1 | 95.475 | 1.9211 | 0.168969 |  |
| Group | 0.0001788 | 0.0001788 | 1 | 95.615 | 0.6470 | 0.423181 |  |
| ADHD:Hemi | 0.0029321 | 0.0029321 | 1 | 99.633 | 10.6099 | 0.001538 | ** |
| Hemi:Age | 0.0016813 | 0.0016813 | 1 | 99.320 | 6.0836 | 0.015356 | * |
| Hemi:Sex | 0.0005330 | 0.0005330 | 1 | 99.310 | 1.9287 | 0.168010 |  |
| Hemi:IQ | 0.0000494 | 0.0000494 | 1 | 99.292 | 0.1788 | 0.673286 |  |
| Hemi:Group | 0.0014778 | 0.0014778 | 1 | 99.332 | 5.3473 | 0.022819 | * |
| --- |  |  |  |  |  |  |  |
| Signif. codes: 0 ‘***’ 0.001 ‘**’ 0.01 ‘*’ 0.05 ‘.’ 0.1 ‘ ’ 1 | | | | | | |  |

**Table B.2 – Association between ‘callous-unemotional’ traits within the case group for Axial Diffusivity in the dorsal CB.**

Type III Analysis of Variance Table with Satterthwaite's method

Sum Sq Mean Sq NumDF DenDF F value Pr(>F)

ADHD 0.00000000 0.00000000 1 43.231 0.0000 0.999524

hemi 0.00003263 0.00003263 1 48.000 0.0848 0.772164

Age 0.00282255 0.00282255 1 43.884 7.3345 0.009606 **

Sex 0.00012554 0.00012554 1 43.240 0.3262 0.570847

IQ 0.00028230 0.00028230 1 43.143 0.7336 0.396463

CU 0.00025800 0.00025800 1 43.457 0.6704 0.417369

ADHD:hemi 0.00062390 0.00062390 1 48.000 1.6212 0.209051

hemi:Age 0.00036169 0.00036169 1 48.000 0.9399 0.337173

hemi:Sex 0.00036396 0.00036396 1 48.000 0.9458 0.335674

hemi:IQ 0.00029315 0.00029315 1 48.000 0.7618 0.387123

hemi:CU 0.00093659 0.00093659 1 48.000 2.4338 0.125316

---

Signif. codes: 0 ‘***’ 0.001 ‘**’ 0.01 ‘*’ 0.05 ‘.’ 0.1 ‘ ’ 1

**Table B.3 – Association between ‘callous-unemotional’ traits within the case group for Mean Diffusivity in the dorsal CB.**

Type III Analysis of Variance Table with Satterthwaite's method

Sum Sq Mean Sq NumDF DenDF F value Pr(>F)

ADHD 0.0000151 0.0000151 1 43.214 0.0737 0.78731

hemi 0.0006290 0.0006290 1 48.000 3.0610 0.08658 .

Age 0.0051795 0.0051795 1 43.898 25.2055 9.055e-06 ***

Sex 0.0000338 0.0000338 1 43.224 0.1643 0.68724

IQ 0.0006197 0.0006197 1 43.121 3.0155 0.08961 .

CU 0.0000147 0.0000147 1 43.450 0.0715 0.79042

ADHD:hemi 0.0000311 0.0000311 1 48.000 0.1516 0.69876

hemi:Age 0.0003375 0.0003375 1 48.000 1.6424 0.20616

hemi:Sex 0.0000004 0.0000004 1 48.000 0.0022 0.96305

hemi:IQ 0.0011573 0.0011573 1 48.000 5.6317 0.02169 *

hemi:CU 0.0005510 0.0005510 1 48.000 2.6816 0.10805

---

Signif. codes: 0 ‘***’ 0.001 ‘**’ 0.01 ‘*’ 0.05 ‘.’ 0.1 ‘ ’ 1

**Table B.4 – Association between ‘callous-unemotional’ traits within the case group for Fractional Anisotropy in the dorsal CB.**

Type III Analysis of Variance Table with Satterthwaite's method

Sum Sq Mean Sq NumDF DenDF F value Pr(>F)

ADHD 0.00000023 0.00000023 1 46.071 0.0015 0.969070

hemi 0.00143291 0.00143291 1 48.001 9.6763 0.003139 **

Age 0.00101844 0.00101844 1 47.982 6.8775 0.011664 *

Sex 0.00001907 0.00001907 1 46.550 0.1288 0.721338

IQ 0.00010442 0.00010442 1 45.105 0.7052 0.405480

CU 0.00017607 0.00017607 1 47.215 1.1890 0.281074

ADHD:hemi 0.00013510 0.00013510 1 48.001 0.9123 0.344292

hemi:Age 0.00007363 0.00007363 1 48.001 0.4972 0.484124

hemi:Sex 0.00023990 0.00023990 1 48.001 1.6200 0.209221

hemi:IQ 0.00089441 0.00089441 1 48.001 6.0399 0.017649 *

hemi:CU 0.00000643 0.00000643 1 48.001 0.0434 0.835872

---

Signif. codes: 0 ‘***’ 0.001 ‘**’ 0.01 ‘*’ 0.05 ‘.’ 0.1 ‘ ’ 1

**Table B.5 – Association between ‘callous-unemotional’ traits within the case group for Radial Diffusivity in the dorsal CB.**

Type III Analysis of Variance Table with Satterthwaite's method

Sum Sq Mean Sq NumDF DenDF F value Pr(>F)

ADHD 0.0000393 0.0000393 1 43.510 0.1748 0.677949

hemi 0.0016383 0.0016383 1 48.000 7.2858 0.009568 **

Age 0.0063357 0.0063357 1 45.258 28.1760 3.231e-06 ***

Sex 0.0000050 0.0000050 1 43.603 0.0222 0.882259

IQ 0.0008759 0.0008759 1 43.242 3.8955 0.054827 .

CU 0.0000140 0.0000140 1 44.162 0.0622 0.804154

ADHD:hemi 0.0000170 0.0000170 1 48.000 0.0754 0.784811

hemi:Age 0.0003257 0.0003257 1 48.000 1.4485 0.234674

hemi:Sex 0.0000729 0.0000729 1 48.000 0.3241 0.571779

hemi:IQ 0.0018034 0.0018034 1 48.000 8.0202 0.006741 **

hemi:CU 0.0003964 0.0003964 1 48.000 1.7628 0.190556

---

Signif. codes: 0 ‘***’ 0.001 ‘**’ 0.01 ‘*’ 0.05 ‘.’ 0.1 ‘ ’ 1

**Table B.6 – Association between ‘callous-unemotional’ traits within the case group for Axial Diffusivity in the ventral CB.**

Type III Analysis of Variance Table with Satterthwaite's method

Sum Sq Mean Sq NumDF DenDF F value Pr(>F)

ADHD 0.0017267 0.0017267 1 43.354 0.8073 0.373876

hemi 0.0004808 0.0004808 1 48.000 0.2248 0.637538

Age 0.0201645 0.0201645 1 44.369 9.4282 0.003641 **

Sex 0.0006817 0.0006817 1 43.381 0.3187 0.575284

IQ 0.0012544 0.0012544 1 43.211 0.5865 0.447926

CU 0.0008800 0.0008800 1 43.714 0.4114 0.524590

ADHD:hemi 0.0086690 0.0086690 1 48.000 4.0533 0.049710 *

hemi:Age 0.0059285 0.0059285 1 48.000 2.7720 0.102442

hemi:Sex 0.0000059 0.0000059 1 48.000 0.0027 0.958491

hemi:IQ 0.0000586 0.0000586 1 48.000 0.0274 0.869253

hemi:CU 0.0003332 0.0003332 1 48.000 0.1558 0.694827

---

Signif. codes: 0 ‘***’ 0.001 ‘**’ 0.01 ‘*’ 0.05 ‘.’ 0.1 ‘ ’ 1

**Table B.7 – Association between ‘callous-unemotional’ traits within the case group for Mean Diffusivity in the ventral CB.**

Type III Analysis of Variance Table with Satterthwaite's method

Sum Sq Mean Sq NumDF DenDF F value Pr(>F)

ADHD 0.0000192 0.0000192 1 43.457 0.0413 0.83996

hemi 0.0005207 0.0005207 1 48.000 1.1221 0.29476

Age 0.0095140 0.0095140 1 44.675 20.5028 4.396e-05 ***

Sex 0.0000553 0.0000553 1 43.500 0.1193 0.73150

IQ 0.0019720 0.0019720 1 43.282 4.2498 0.04529 *

CU 0.0001621 0.0001621 1 43.895 0.3492 0.55757

ADHD:hemi 0.0031858 0.0031858 1 48.000 6.8654 0.01173 *

hemi:Age 0.0023878 0.0023878 1 48.000 5.1457 0.02784 *

hemi:Sex 0.0001598 0.0001598 1 48.000 0.3443 0.56012

hemi:IQ 0.0001863 0.0001863 1 48.000 0.4014 0.52937

hemi:CU 0.0000007 0.0000007 1 48.000 0.0014 0.97016

---

Signif. codes: 0 ‘***’ 0.001 ‘**’ 0.01 ‘*’ 0.05 ‘.’ 0.1 ‘ ’ 1

**Table B.8 – Association between ‘callous-unemotional’ traits within the case group for Fractional Anisotropy in the ventral CB.**

Type III Analysis of Variance Table with Satterthwaite's method

Sum Sq Mean Sq NumDF DenDF F value Pr(>F)

ADHD 0.0012272 0.0012272 1 46.301 1.7738 0.18943

hemi 0.0000560 0.0000560 1 48.000 0.0809 0.77730

Age 0.0043855 0.0043855 1 47.892 6.3390 0.01521 *

Sex 0.0010926 0.0010926 1 46.794 1.5792 0.21511

IQ 0.0028843 0.0028843 1 45.209 4.1690 0.04703 *

CU 0.0001759 0.0001759 1 47.399 0.2542 0.61646

ADHD:hemi 0.0004008 0.0004008 1 48.000 0.5793 0.45031

hemi:Age 0.0001121 0.0001121 1 48.000 0.1620 0.68913

hemi:Sex 0.0003321 0.0003321 1 48.000 0.4800 0.49178

hemi:IQ 0.0001344 0.0001344 1 48.000 0.1942 0.66142

hemi:CU 0.0001156 0.0001156 1 48.000 0.1670 0.68458

---

Signif. codes: 0 ‘***’ 0.001 ‘**’ 0.01 ‘*’ 0.05 ‘.’ 0.1 ‘ ’ 1

**Table B.9 – Association between ‘callous-unemotional’ traits within the case group for Radial Diffusivity in the ventral CB.**

Type III Analysis of Variance Table with Satterthwaite's method

Sum Sq Mean Sq NumDF DenDF F value Pr(>F)

ADHD 0.0000558 0.0000558 1 43.998 0.1777 0.67545

hemi 0.0005412 0.0005412 1 48.000 1.7225 0.19561

Age 0.0073891 0.0073891 1 46.039 23.5168 1.452e-05 ***

Sex 0.0003566 0.0003566 1 44.142 1.1349 0.29253

IQ 0.0024266 0.0024266 1 43.658 7.7229 0.00801 **

CU 0.0000683 0.0000683 1 44.789 0.2173 0.64339

ADHD:hemi 0.0014524 0.0014524 1 48.000 4.6225 0.03662 *

hemi:Age 0.0012110 0.0012110 1 48.000 3.8540 0.05544 .

hemi:Sex 0.0003150 0.0003150 1 48.000 1.0026 0.32169

hemi:IQ 0.0002771 0.0002771 1 48.000 0.8818 0.35241

hemi:CU 0.0001069 0.0001069 1 48.000 0.3403 0.56236

---

Signif. codes: 0 ‘***’ 0.001 ‘**’ 0.01 ‘*’ 0.05 ‘.’ 0.1 ‘ ’ 1

**Table B.10 – Association between ‘callous-unemotional’ traits within the case group for Axial Diffusivity in the UF.**

Type III Analysis of Variance Table with Satterthwaite's method

Sum Sq Mean Sq NumDF DenDF F value Pr(>F)

ADHD 0.0000761 0.0000761 1 43.484 0.1336 0.7165236

hemi 0.0018254 0.0018254 1 47.357 3.2060 0.0797607 .

Age 0.0088581 0.0088581 1 43.419 15.5579 0.0002877 ***

Sex 0.0000007 0.0000007 1 43.108 0.0012 0.9719931

IQ 0.0012025 0.0012025 1 43.452 2.1121 0.1533263

CU 0.0002936 0.0002936 1 43.150 0.5156 0.4765966

ADHD:hemi 0.0007210 0.0007210 1 47.727 1.2663 0.2660800

hemi:Age 0.0039078 0.0039078 1 47.164 6.8634 0.0117982 *

hemi:Sex 0.0001137 0.0001137 1 47.235 0.1997 0.6569867

hemi:IQ 0.0003059 0.0003059 1 47.748 0.5373 0.4671193

hemi:CU 0.0006458 0.0006458 1 47.162 1.1343 0.2922811

---

Signif. codes: 0 ‘***’ 0.001 ‘**’ 0.01 ‘*’ 0.05 ‘.’ 0.1 ‘ ’ 1

**Table B.11 – Association between ‘callous-unemotional’ traits within the case group for Mean Diffusivity in the UF.**

Type III Analysis of Variance Table with Satterthwaite's method

Sum Sq Mean Sq NumDF DenDF F value Pr(>F)

ADHD 0.00000022 0.00000022 1 43.544 0.0011 0.9742424

hemi 0.00057251 0.00057251 1 47.322 2.7071 0.1065317

Age 0.00311805 0.00311805 1 43.768 14.7433 0.0003929 ***

Sex 0.00002837 0.00002837 1 43.259 0.1341 0.7159595

IQ 0.00041281 0.00041281 1 43.481 1.9519 0.1694708

CU 0.00001524 0.00001524 1 43.382 0.0721 0.7896271

ADHD:hemi 0.00094047 0.00094047 1 47.604 4.4469 0.0402520 *

hemi:Age 0.00078566 0.00078566 1 47.174 3.7149 0.0599619 .

hemi:Sex 0.00003342 0.00003342 1 47.229 0.1580 0.6927728

hemi:IQ 0.00003129 0.00003129 1 47.621 0.1479 0.7022217

hemi:CU 0.00000870 0.00000870 1 47.173 0.0411 0.8401341

---

Signif. codes: 0 ‘***’ 0.001 ‘**’ 0.01 ‘*’ 0.05 ‘.’ 0.1 ‘ ’ 1

**Table B.12 – Association between ‘callous-unemotional’ traits within the case group for Fractional Anisotropy in the UF.**

Type III Analysis of Variance Table with Satterthwaite's method

Sum Sq Mean Sq NumDF DenDF F value Pr(>F)

ADHD 0.00005992 0.00005992 1 45.050 0.2434 0.6242

hemi 0.00004900 0.00004900 1 47.639 0.1990 0.6575

Age 0.00019340 0.00019340 1 46.639 0.7855 0.3800

Sex 0.00011965 0.00011965 1 44.710 0.4859 0.4894

IQ 0.00005315 0.00005315 1 44.651 0.2159 0.6445

CU 0.00040314 0.00040314 1 45.287 1.6373 0.2072

ADHD:hemi 0.00025936 0.00025936 1 48.168 1.0534 0.3099

hemi:Age 0.00035254 0.00035254 1 47.365 1.4318 0.2374

hemi:Sex 0.00000577 0.00000577 1 47.466 0.0234 0.8790

hemi:IQ 0.00003121 0.00003121 1 48.199 0.1268 0.7234

hemi:CU 0.00019382 0.00019382 1 47.363 0.7872 0.3794

**Table B.13 – Association between ‘callous-unemotional’ traits within the case group for Radial Diffusivity in the UF.**

Type III Analysis of Variance Table with Satterthwaite's method

Sum Sq Mean Sq NumDF DenDF F value Pr(>F)

ADHD 0.00002464 0.00002464 1 43.850 0.1070 0.745165

hemi 0.00020753 0.00020753 1 47.412 0.9012 0.347276

Age 0.00238499 0.00238499 1 44.524 10.3569 0.002408 **

Sex 0.00007542 0.00007542 1 43.549 0.3275 0.570065

IQ 0.00030740 0.00030740 1 43.713 1.3349 0.254216

CU 0.00000152 0.00000152 1 43.822 0.0066 0.935530

ADHD:hemi 0.00107755 0.00107755 1 47.734 4.6793 0.035567 *

hemi:Age 0.00011614 0.00011614 1 47.243 0.5043 0.481095

hemi:Sex 0.00001063 0.00001063 1 47.305 0.0461 0.830834

hemi:IQ 0.00000033 0.00000033 1 47.753 0.0014 0.969917

hemi:CU 0.00006866 0.00006866 1 47.242 0.2982 0.587609

---

Signif. codes: 0 ‘***’ 0.001 ‘**’ 0.01 ‘*’ 0.

**Table B.14 – Association between ‘callous-unemotional’ and primary and secondary CU traits within the case group for Axial Diffusivity in the dorsal CB.**

Type III Analysis of Variance Table with Satterthwaite's method

Sum Sq Mean Sq NumDF DenDF F value Pr(>F)

ADHD 0.00068588 0.00068588 1 30.298 1.5615 0.22101

hemi 0.00034386 0.00034386 1 34.000 0.7828 0.38249

Age 0.00017234 0.00017234 1 30.691 0.3924 0.53569

Sex 0.00018212 0.00018212 1 30.433 0.4146 0.52445

IQ 0.00084397 0.00084397 1 30.629 1.9214 0.17571

Pr/Sec CU 0.00141612 0.00141612 1 30.479 3.2240 0.08249 .

CU 0.00121579 0.00121579 1 30.351 2.7679 0.10646

ADHD:hemi 0.00030305 0.00030305 1 34.000 0.6899 0.41198

hemi:Age 0.00073771 0.00073771 1 34.000 1.6795 0.20372

hemi:Sex 0.00049730 0.00049730 1 34.000 1.1322 0.29481

hemi:IQ 0.00057385 0.00057385 1 34.000 1.3065 0.26102

hemi:Pr/Sec CU 0.00231950 0.00231950 1 34.000 5.2807 0.02784 *

Pr/Sec CU:CU 0.00146990 0.00146990 1 30.404 3.3465 0.07717 .

hemi:CU 0.00007311 0.00007311 1 34.000 0.1664 0.68586

hemi:Pr/Sec CU:CU 0.00273835 0.00273835 1 34.000 6.2343 0.01754 *

---

Signif. codes: 0 ‘***’ 0.001 ‘**’ 0.01 ‘*’ 0.05 ‘.’ 0.1 ‘ ’ 1

**Table B.15 – Association between the ‘unemotional’ ICU subscale and primary and secondary CU traits within the case group for Axial Diffusivity in the dorsal CB.**

Type III Analysis of Variance Table with Satterthwaite's method

Sum Sq Mean Sq NumDF DenDF F value Pr(>F)

ADHD 0.0008341 0.0008341 1 30.523 2.1498 0.152819

hemi 0.0003991 0.0003991 1 34.000 1.0287 0.317632

Age 0.0000619 0.0000619 1 30.835 0.1595 0.692385

Sex 0.0000274 0.0000274 1 30.412 0.0705 0.792351

IQ 0.0006417 0.0006417 1 30.599 1.6538 0.208087

Pr/Sec CU 0.0016620 0.0016620 1 30.987 4.2836 0.046907 *

Unemotional 0.0003794 0.0003794 1 30.567 0.9778 0.330497

ADHD:hemi 0.0017687 0.0017687 1 34.000 4.5586 0.040044 *

hemi:Age 0.0014471 0.0014471 1 34.000 3.7295 0.061830 .

hemi:Sex 0.0000287 0.0000287 1 34.000 0.0739 0.787361

hemi:IQ 0.0005269 0.0005269 1 34.000 1.3580 0.251988

hemi: Pr/Sec CU 0.0029141 0.0029141 1 34.000 7.5107 0.009703 **

Pr/Sec CU:Unemotional 0.0018784 0.0018784 1 30.806 4.8412 0.035416 *

hemi:Unemotional 0.0000628 0.0000628 1 34.000 0.1618 0.690002

hemi:Pr/Sec CU:Unemotional 0.0041839 0.0041839 1 34.000 10.7833 0.002377 **

---

Signif. codes: 0 ‘***’ 0.001 ‘**’ 0.01 ‘*’ 0.05 ‘.’ 0.1 ‘ ’ 1

**Figure A.1 – Association between the ‘uncaring’ ICU subscale, primary versus secondary CU traits and left versus right hemispheres for axial diffusivity in the dorsal CB (*p .252)*.**

**
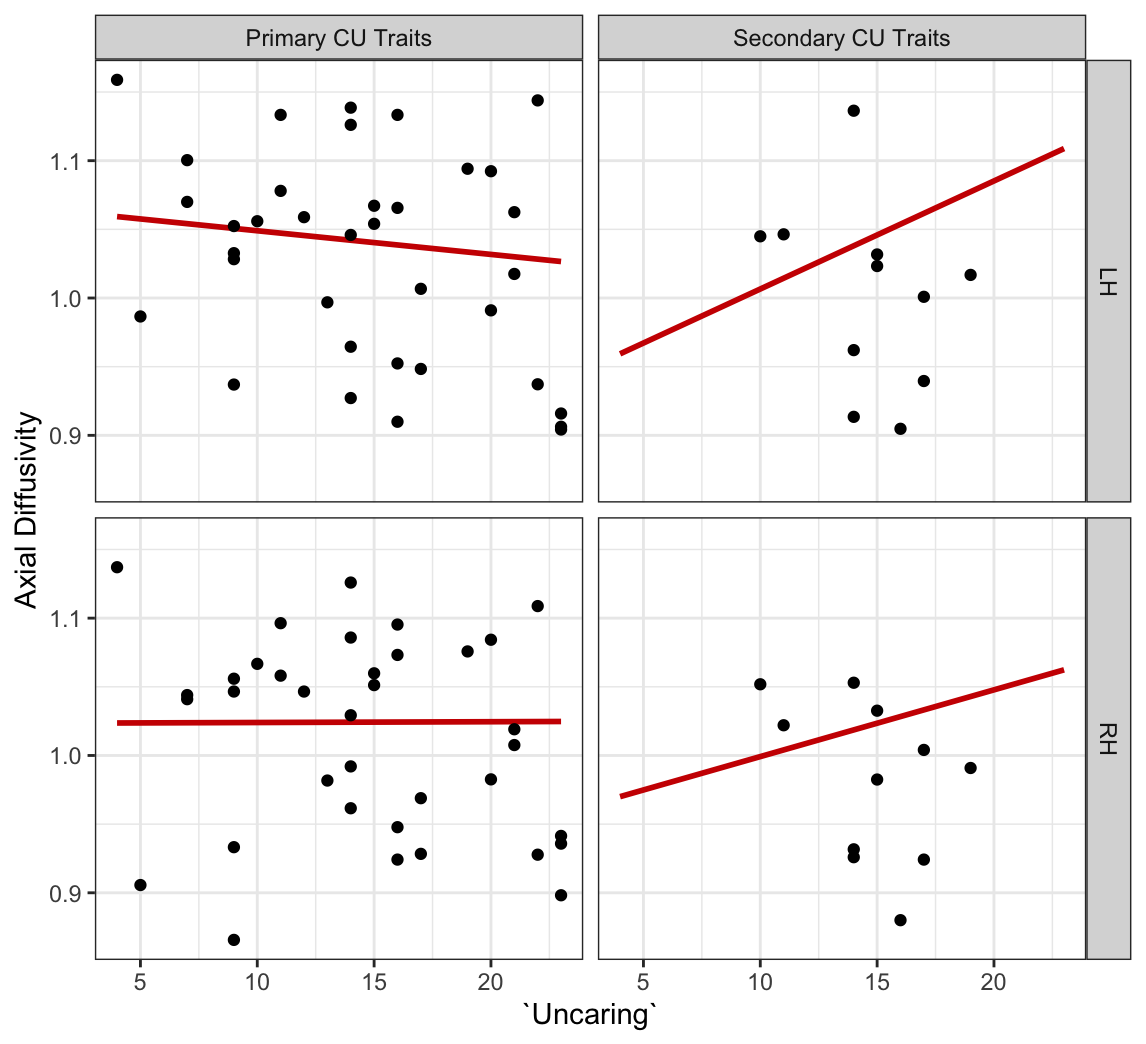
**

**Figure A.2 – Association between the ‘callousness’ ICU subscale, primary versus secondary CU traits and left versus right hemispheres for axial diffusivity in the dorsal CB (*p .285)*.**

**
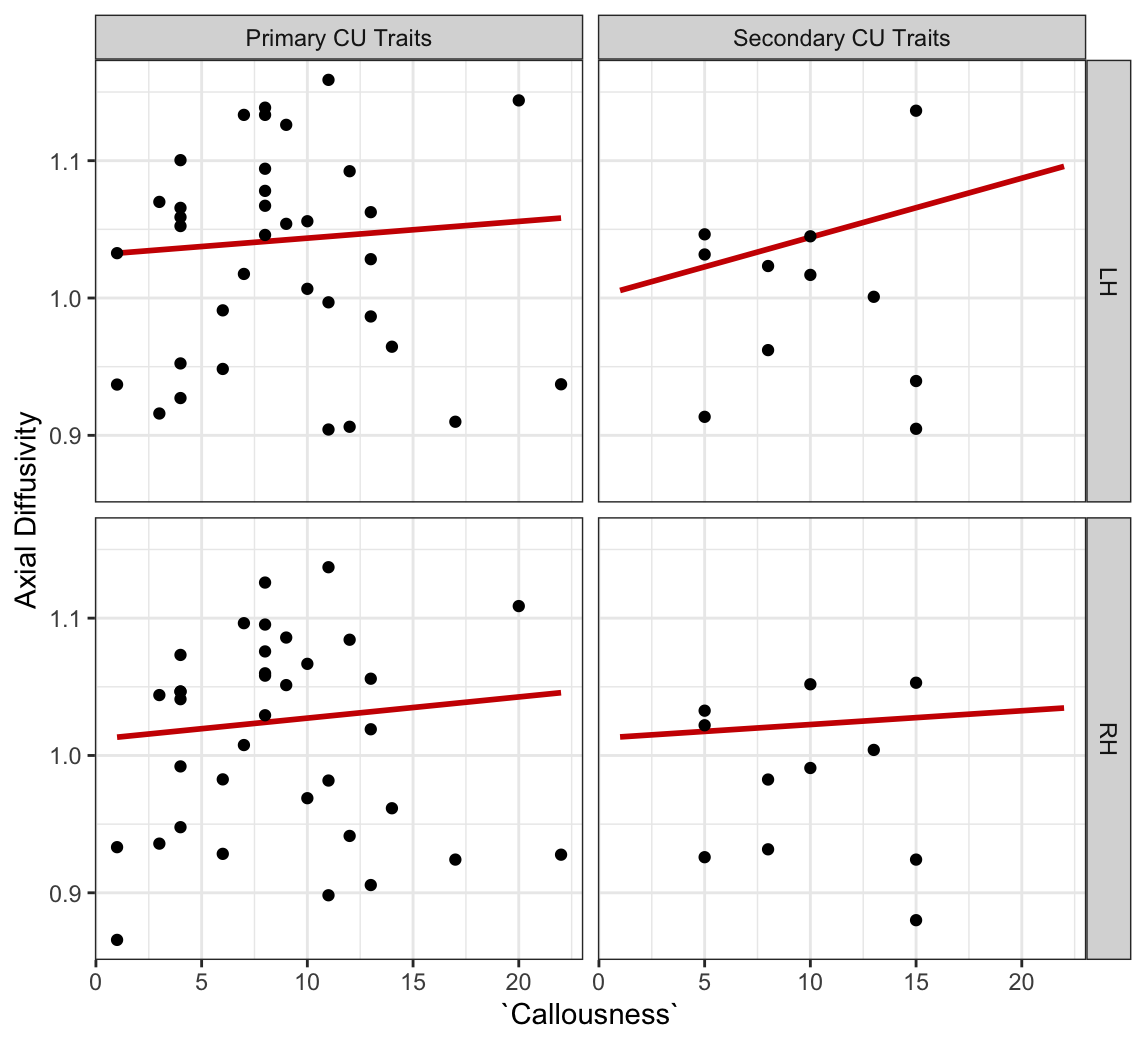
**

**Table C.1 – Association between the ‘unemotional’ ICU subscale and primary and secondary CU traits within the case group for Mean Diffusivity in the dorsal CB.**

Type III Analysis of Variance Table with Satterthwaite's method

Sum Sq Mean Sq NumDF DenDF F value Pr(>F)

ADHD. 0.00006019 0.00006019 1 30.729 0.2756 0.6033688

hemi 0.00154130 0.00154130 1 34.000 7.0573 0.0119344 *

Age 0.00292355 0.00292355 1 31.200 13.3864 0.0009275 ***

Sex 0.00005008 0.00005008 1 30.576 0.2293 0.6354335

IQ 0.00058158 0.00058158 1 30.820 2.6630 0.1128872

Pr/Sec Cu 0.00020032 0.00020032 1 31.424 0.9172 0.3455139

Unemotional 0.00038596 0.00038596 1 30.816 1.7672 0.1934810

ADHD:hemi 0.00041394 0.00041394 1 34.000 1.8954 0.1775966

hemi:Age 0.00073112 0.00073112 1 34.000 3.3477 0.0760802 .

hemi:Sex 0.00005594 0.00005594 1 34.000 0.2562 0.6160379

hemi:IQ 0.00150149 0.00150149 1 34.000 6.8751 0.0129827 *

hemi:Pr/Sec CU 0.00089477 0.00089477 1 34.000 4.0970 0.0508737 .

Pr/Sec Cu:Unemotional 0.00015173 0.00015173 1 31.159 0.6947 0.4109015

hemi:Unemotional 0.00000001 0.00000001 1 34.000 0.0000 0.9954227

hemi:Pr/Sec CU:Unemotional 0.00139642 0.00139642 1 34.000 6.3940 0.0162594 *

---

Signif. codes: 0 ‘***’ 0.001 ‘**’ 0.01 ‘*’ 0.05 ‘.’ 0.1 ‘ ’ 1

**Figure B.1 – Association between the ‘uncaring’ ICU subscale, primary versus secondary CU traits and left versus right hemispheres for mean diffusivity in the dorsal CB (*p .082)*.**


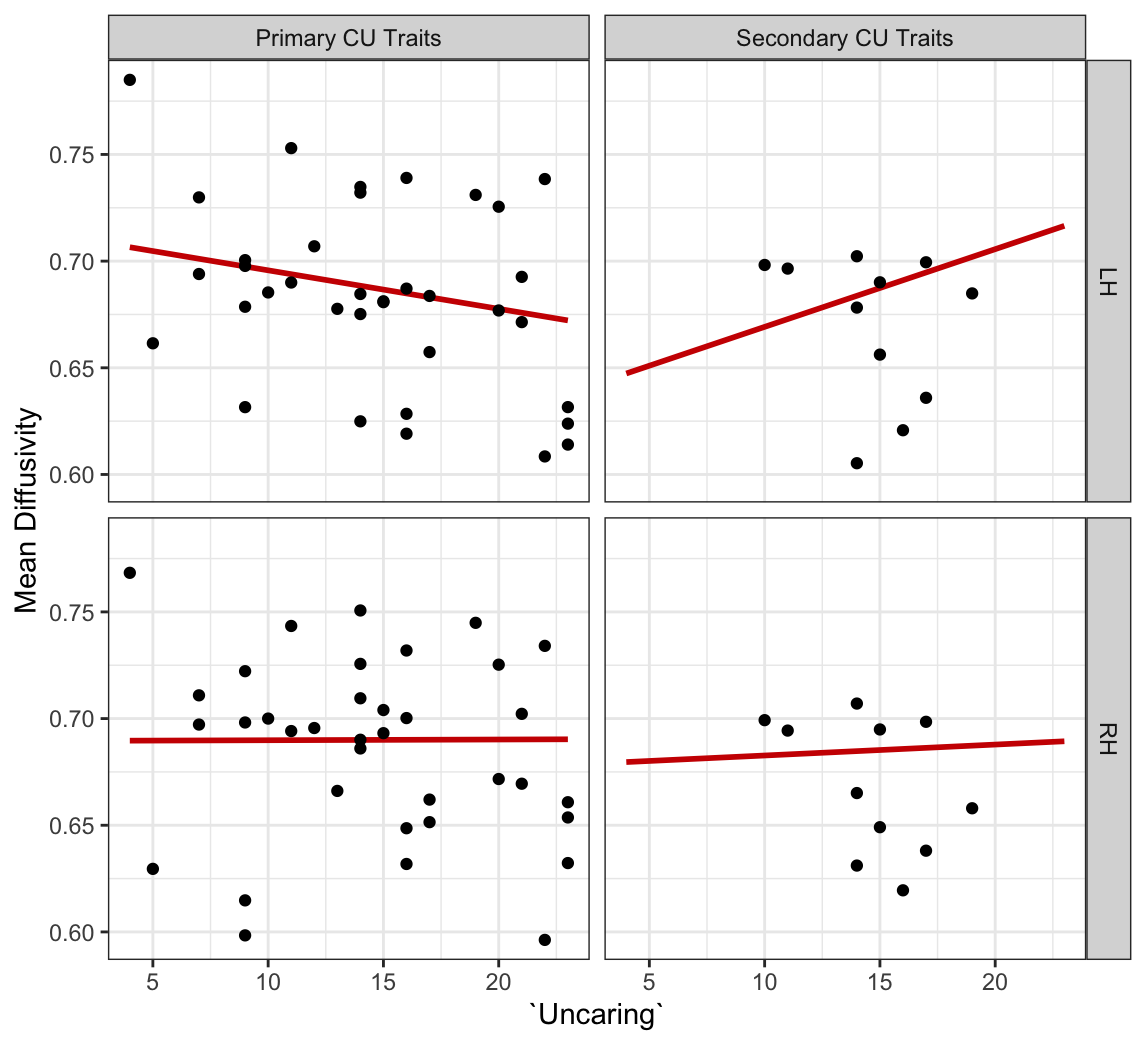


**Figure B.2 – Association between the ‘callousness’ ICU subscale, primary versus secondary CU traits and left versus right hemispheres for mean diffusivity in the dorsal CB (*p .727)*.**


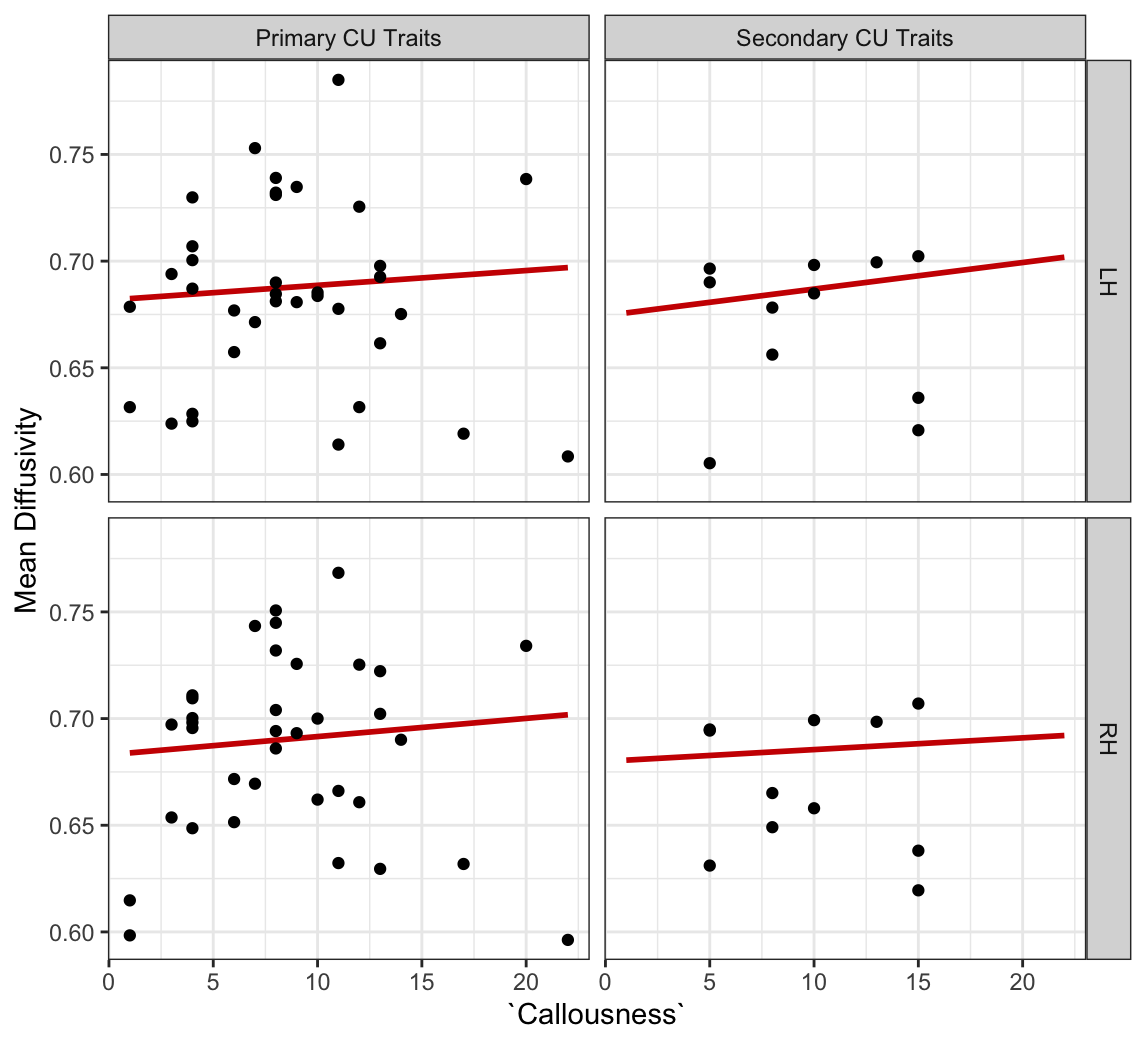

Supplement: Supplementary file 1 — Supplementary Material 1 [file 787_2025_2806_MOESM1_ESM.docx]
